# Elucidating the Neurobiological Underpinnings of Mild Behavioral Impairment in Tauopathies: Clinical and Molecular Insights

**DOI:** 10.3390/ijms27073341

**Published:** 2026-04-07

**Authors:** Efthalia Angelopoulou, John Papatriantafyllou, Sokratis Papageorgiou, Chiara Villa

**Affiliations:** 1 1st Department of Neurology, Aiginition University Hospital, Medical School, National and Kapodistrian University of Athens, 11528 Athens, Greece; angelthal@med.uoa.gr (E.A.); jpapatriantafyllou@gmail.com (J.P.); sokpapa@med.uoa.gr (S.P.); 2 Third Age Day Care Center IASIS, 16562 Athens, Greece; 3 School of Medicine and Surgery, University of Milano-Bicocca, 20900 Monza, Italy

**Keywords:** mild behavioral impairment, tauopathies, frontotemporal dementia, Alzheimer’s disease, neurodegeneration

## Abstract

Mild behavioral impairment (MBI) is a clinical syndrome characterized by the late-life onset and persistence of neuropsychiatric symptoms (NPSs), representing a change from longstanding behavior or personality and considered a potential prodrome of neurodegenerative disease. MBI is classified into five domains: decreased motivation, affective dysregulation, impulse dyscontrol, social inappropriateness, and psychotic symptoms. In this narrative review, we synthesize clinical, neuroanatomical, and molecular evidence linking MBI to the spectrum of tauopathies, including Alzheimer’s disease (AD), frontotemporal spectrum disorders (FTSDs), and primary four-repeat tauopathies such as progressive supranuclear palsy (PSP) and corticobasal degeneration (CBD). Emerging evidence suggests that early behavioral symptoms associated with MBI may reflect the selective vulnerability of frontolimbic, salience, default mode, and frontostriatal networks to tau-mediated neurodegeneration. Mechanistically, converging findings support roles for tau-related synaptic dysfunction, including synaptotoxic soluble tau species, cytoskeletal and axonal transport disruption, monoaminergic neurotransmitter imbalance in brainstem systems, and neuroinflammatory and glial pathways. We also highlight genotype-related behavioral profiles in genetic frontotemporal lobar degeneration and discuss how scalable blood-based biomarkers, including neurofilament light chain, glial fibrillary acidic protein, and plasma phospho-tau species, may complement MBI-based phenotyping for differential diagnosis and prognostic stratification in clinical research.

## 1. Introduction

Mild behavioral impairment (MBI) is a syndrome characterized by later-life onset and the persistence of neuropsychiatric symptoms (NPSs), representing a potential early manifestation of neurodegenerative diseases [1,2]. The concept of MBI was developed to capture the neurobehavioral axis of prodromal neurodegeneration that can emerge with or without objective cognitive impairment, complementing the construct of mild cognitive impairment (MCI) [1,2]. NPSs, such as depressive symptoms, anxiety, apathy, irritability, loss of empathy and psychotic manifestations, are common across neurodegenerative disorders and are increasingly recognized as an integral part of the disease, potentially representing early clinical signs of the underlying neurodegenerative process [3]. In this regard, MBI provides a standardized framework to appropriately capture these early behavioral changes [2]. The recent MBI criteria highlight some core features that help distinguish MBI-related NPSs from NPSs in dementia or primary psychiatric conditions, emphasizing the onset in later life, the persistence, and the clear change from longstanding baseline behavior, thereby improving the signal-to-noise ratio when studying prodromal disease [2].

Tauopathies comprise a heterogeneous group of neurodegenerative diseases defined by the abnormal aggregation of the tau protein, spanning Alzheimer’s disease (AD) and primary tauopathies such as progressive supranuclear palsy (PSP), corticobasal degeneration (CBD), and frontotemporal lobar degeneration with tau pathology (FTLD-tau) [4]. Despite their marked clinicopathological overlap, tauopathies show, at least partially, some distinct patterns of network degeneration and neuropsychiatric phenotypes, including apathy, affective dysregulation, impulsivity and disinhibition, as well as changes in social cognition [4], which may potentially map onto MBI domains. The concept of domain-based mapping is attractive because it allows behavioral symptoms to be studied as structured clinical outcomes linked to large-scale neural networks, rather than as nonspecific psychiatric comorbidities. Importantly, the current biomarker era is altering how prodromal neurodegenerative syndromes are defined and studied. While plasma, cerebrospinal fluid (CSF) and neuroimaging tools are rapidly evolving for AD, biomarker translation for primary tauopathies remains a critical unmet need, particularly for participant enrichment in clinical trials at the early stages of the diseases [4]. Recent translational advances, including tau species that may support discrimination among primary tauopathies in CSF, highlight the importance of a biologically based diagnosis. Within this emerging landscape, MBI assessment could function as a scalable clinical tool that could complement biomarker strategies by identifying individuals who might be more likely to harbor evolving neurodegenerative pathology. Emerging evidence also suggests that specific MBI domains may show longitudinal associations with plasma markers relevant to AD pathophysiology, including phosphorylated tau and potentially broader metabolic or neurodegenerative signatures [5]. Although the potential underlying pathophysiology of MBI in AD has been already reviewed [5,6,7], whether and how these relationships can be generalized, especially across the whole spectrum of tauopathies, remains largely underexplored.

In this narrative review, given the growing evidence of MBI in prodromal neurodegeneration, we examine MBI as a transdiagnostic construct across tauopathies, focusing on the clinician and mechanistic links between tau-mediated network degeneration and emergent behavioral changes. For this purpose, we synthesize current knowledge on MBI across the spectrum of tauopathies, encompassing clinical, neuroanatomical, and molecular insights, highlighting both shared and disease-specific features, aiming to position MBI as a useful link between clinical phenotyping and molecular neurodegeneration.

## 2. Methods

To identify relevant evidence, we followed a structured literature search approach. We searched MEDLINE and Scopus databases with no a priori time restrictions, using combinations of controlled vocabulary terms and free-text keywords related to the following keywords: “mild behavioral impairment”, “neuropsychiatric symptoms”, individual MBI domains including “apathy”, “depression”, “anxiety”, “irritability”, “agitation”, “disinhibition”, “compulsivity”, “psychosis”, “hallucinations”, “delusions”, and tau-related terms, including “tauopathy”, “tau proteinopathy”, “tau”, “Alzheimer’s disease”, “frontotemporal dementia”, “behavioral variant FTD”, “progressive supranuclear palsy”, “corticobasal degeneration”, and “Pick’s disease”. Search results were screened at the title and abstract level, and potentially relevant full texts were reviewed. We primarily included clinical studies investigating MBI in tauopathies using MBI-C or compatible operational definitions, as well as preclinical studies elucidating potential mechanisms linking tau-mediated network or cellular dysfunction to behavioral phenotypes consistent with MBI domains, including synaptic dysfunction, neurotransmitter alterations and neuroinflammatory pathways. For discussion purposes, we included additional studies reporting NPSs in the broader context of preclinical or prodromal stages of neurodegenerative diseases when directly informative for tauopathy–MBI relationships. The reference lists of the included articles were manually screened with a snowball process to identify additional eligible studies. Given the expected heterogeneity in designs, instruments, and outcomes, evidence was synthesized narratively, organized in two levels: (i) tauopathy clinical syndrome; (ii) convergent molecular and network-related mechanisms linking tau pathology to early behavioral changes.

## 3. MBI Definition

MBI is a neurobehavioral syndrome proposed to capture the late-life emergence of persistent NPSs as an at-risk state for incident cognitive decline and dementia, with or without concurrent cognitive complaints. Although the concept has earlier roots in observations of prodromal behavioral change particularly in frontotemporal spectrum disorders (FTSDs), the recent research framework for MBI was defined by the International Society to Advance Alzheimer’s Research and Treatment: Alzheimer’s Association (ISTAART-AA), Neuropsychiatric Syndromes Professional Interest Area (PIA) in 2016 [2]. According to these criteria, MBI is characterized by the onset of behavioral or personality changes present, for at least 6 months, after the age of 50 years; the behavioral changes must represent a deviation from an individual’s usual behavior or personality and must not be better explained by a primary psychiatric diagnosis, a medical condition or substance effects [2].

Importantly, MBI may coexist with normal cognition, subjective cognitive decline (SCD), or MCI, but it is conceptually distinct from MCI and should not be viewed as a competing construct [2]. NPSs in the context of MBI must be directly associated with at least mild functional impairment, though not severe enough to meet the diagnostic criteria for dementia. MBI should be distinguished from both classical psychiatric disorders (major depression, bipolar disorder, general anxiety disorder, delusional disorder, schizophrenia, etc.) and the traditional behavioral and psychological symptoms of dementia (BPSD), as seen in established dementia [2]. MBI can be considered as the predementia behavioral axis that parallels the cognitive axis of neurodegeneration that is mainly captured by MCI [1,2].

A key strength of the MBI construct lies in its attempt to improve the specificity of the assessment of NPSs in older adults. Conventional neuropsychiatric frameworks often capture symptoms that are transient, reactive, or attributable to life stressors, whereas MBI requires later-life onset, persistence, and clear change from baseline. This distinction is particularly important in aging populations, in whom depression, anxiety, irritability, apathy, or suspiciousness may arise for multiple reasons unrelated to neurodegeneration. By requiring persistence for at least 6 months and excluding longstanding or recurrent psychiatric syndromes, MBI enriches for behavioral symptoms more likely to reflect evolving brain disease rather than nonspecific psychological distress [2].

The MBI syndrome is organized into five domains: (a) decreased motivation (apathy, reduced initiative), (b) emotional dysregulation (anxiety, depression, mood lability), (c) impulse dyscontrol (agitation, aggression, compulsivity), (d) social inappropriateness (disinhibition, lack of empathy), and (e) abnormal perception or thought content (hallucinations, delusions) [2]. This domain-based approach is especially relevant for tauopathies where different patterns of frontolimbic, salience network, and frontostriatal involvement might potentially underly distinct MBI presentations [8,9]. The operationalization of MBI in research and clinical settings has been greatly facilitated by the development of the MBI Checklist (MBI-C) [1]. The MBI-C is a 34-item instrument specifically developed for predementia populations, designed to directly align with the ISTAART-AA criteria. This is a major methodological advantage over instruments such as the Neuropsychiatric Inventory (NPI), originally developed for dementia populations with shorter reference windows [1]. MBI-C can potentially be used to detect behavioral changes across a wide range of neurodegenerative disorders. For instance, in Parkinson’s disease (PD), MBI-C was recently revised into a 24-item model to prevent floor effects and better capture the smallest manifestations of MBI in PD [10]. Behavioral abnormalities and cognitive impairments, especially executive dysfunctions, can appear early in amyotrophic lateral sclerosis (ALS) and are linked with faster disease progression, lower survival, and increased caregiver burden [11]. Also in this context, MBI was revealed to be a reliable clinical marker of cognitive deterioration both in ALS and other motor neuron diseases (MNDs), showing great potential for identifying dementia in its prodromal or preclinical stages [12,13].

It has been demonstrated that the MBI-C can be utilized across different clinical and research settings. In cognitive clinic cohorts, higher MBI-C scores have been associated with lower cognitive performance, and MBI prevalence increases across SCD, MCI, and dementia groups [14]. In SCD populations, the MBI-C was able to detect subtle but clinically relevant NPSs, and its total score showed significant associations with established neuropsychiatric and depressive symptom measures, including the NPI-Questionnaire (NPI-Q) and the Geriatric Depression Scale-15 (GDS-15), supporting its construct validity in early, predementia stages [15]. Collectively, these findings support the use of the MBI-C as the current standard instrument for MBI ascertainment in both research and increasingly in prodromal clinical phenotyping.

Growing longitudinal evidence further supports the prognostic significance of MBI. In older adults without dementia, MBI has been associated with worse baseline cognition, faster cognitive decline, and increased risk of incident dementia [16]. In a prospective Asian cohort, 38.6% of individuals with MBI developed dementia compared with 12.3% of those without MBI, corresponding to a 2.56-fold increased risk of incident dementia [16]. Similarly, in older adults without dementia from ADNI, MBI was associated with poorer cognition, greater and faster increase in amyloid burden, and an elevated risk of cognitive decline (HR 2.42) [17]. These data suggest that MBI can be a clinically meaningful prognostic construct linked to adverse cognitive trajectories.

Some evidence has also shown connections between genetic background and MBI, indicating that certain MBI domains correlate with different biochemical pathways involved in AD and other dementias. The possibility that MBI domains represent clinically apparent manifestations of underlying genetic susceptibility is highlighted by associations with some genetic variants (Table 1). Among them, the presence of the Apolipoprotein E (*APOE*) ε4 allele, the strongest common genetic risk factor for late-onset AD, was associated with a higher likelihood of affective dysregulation [18]. The same study also reported that variants located on *MS4A4A* and *MS4A6A* genes have been inversely associated with affective dysregulation, while *ZCWPW1* variants have been related to decreased social inappropriateness and psychotic manifestations [18]. *BIN1* and *EPHA1* have been linked to psychosis, whereas *NME8* has been inversely associated with apathy [18]. Other evidence links the presence of least one Met allele in the brain-derived neurotrophic factor (*BDNF*) gene with a higher likelihood of MBI in PD patients, as assessed by MBI-C [19].

Finally, recent work suggests that the way in which MBI is operationalized matters substantially for prognostic accuracy. Comparisons of different case definitions indicate that persistence-based definitions of late-life emergent NPSs outperform transient or nonspecific symptom definitions in dementia risk modeling, reinforcing one of the central conceptual premises of MBI, according to which, sustained behavioral change in later life is more informative than isolated NPSs when attempting to identify prodromal neurodegenerative diseases [20]. The MBI-C has already been translated and validated in multiple languages and settings, supporting its cross-cultural applicability and broader use in both research and clinical practice [21]. In addition, it has shown feasibility for remote administration, including telephone-based assessment in SCD cohorts, further supporting its practicality as a scalable screening tool beyond specialist memory clinics [15]. For these reasons, MBI is increasingly viewed as a scalable clinical enrichment construct that may complement biomarkers in identifying individuals at heightened risk for neurodegenerative disorders, including tauopathies.

## 4. MBI in the Spectrum of Tauopathies: Clinical Insights

### 4.1. MBI in Alzheimer’s Disease: Focusing on Tau Pathology

AD is the most extensively studied tauopathy in relation to MBI and currently provides the strongest evidence linking MBI symptoms with molecular markers of neurodegeneration. AD is characterized by the abnormal accumulation of tau as neurofibrillary tangles (NFTs) and neuropil threads mainly in neurons, as well as in dystrophic neurites accompanied by the aberrant deposition of amyloid-beta plaques extracellularly [22]. While AD has traditionally been conceptualized through its amnestic cognitive presentation, NPSs may emerge early in the disease course, sometimes even before prominent cognitive decline. In biomarker-defined amnestic MCI due to AD (aMCI-AD), approximately half of participants met criteria for MBI (48.4%; 30/62). Compared to 50 cognitively normal older adults, the aMCI-AD group was associated with higher total MBI severity, with the strongest effects in affective dysregulation, apathy, and impulse dyscontrol, whereas social inappropriateness and psychotic symptoms were not significantly different from cognitively normal controls [23].

In larger non-dementia AD spectrum cohorts, MBI has also been associated with poorer cognition and greater pathological burden. In ADNI participants without dementia, MBI was associated with worse global cognition and higher β-amyloid burden. Importantly, amyloid at least partially mediated the association between MBI and cognition, accounting for about 17% of the effect on global cognition and 38% for memory, executive, and language domains [17]. Longitudinally, MBI has been associated with more rapid amyloid accumulation and an elevated risk of cognitive decline [17]. Notably, new tau-focused data suggest that the association is not limited to amyloid. In Aβ-positive ADNI participants with normal cognition or MCI, MBI was associated with greater tau-positron emission tomography (PET) uptake in Braak I and Braak III areas, supporting a link between MBI and early cortical tau deposition in AD-vulnerable regions [24].

Blood-based biomarker studies point in the same direction. In ADNI participants with normal cognition or MCI, MBI was associated cross-sectionally with approximately 8% higher plasma p-tau181 and longitudinally with persistently higher p-tau181, along with decline in memory and executive function [25]. Survival analyses showed a 3.92-fold greater dementia incidence in those with MBI, whereas transient NPSs not meeting MBI criteria were not significantly different from the no-NPS group [25]. Similarly, in the NOLAN cohort, the MBI domain of psychotic features was associated with steeper increases in plasma pTau181 over one year, further supporting the role of tau-mediated pathways underlying MBI within the AD continuum [26]. Findings from CSF studies reinforce this distinction. Compared with no NPSs or transient NPS, MBI has been associated with lower CSF Aβ42 and Aβ42/40 ratios, higher p-tau and t-tau levels, and higher p-tau/Aβ42 and t-tau/Aβ42 ratios, while also conferring substantially greater risk of progression to dementia [27]. Accordingly, MBI was also investigated within the context of the A/T/N framework of AD. In a larger dementia-free cohort, individuals with MBI were correlated with CSF amyloid and tau positivity, as well as with an AD continuum biomarker profile, but not with non-AD pathology [28]. These observations further support the notion that persistent late-life emergent behavioral symptoms are more tightly linked to core AD pathophysiology than nonspecific or fluctuating NPS.

Interestingly, MBI can be proposed as the clinical manifestation of network-level consequences of tau deposition in AD. In this context, recent evidence in participants with aMCI and mild AD suggests that tau positivity might be linked to MBI indirectly through reduced salience network segregation, rather than through a simple direct biomarker-to-symptom association [9]. These findings suggest that emergent behavioral symptoms may arise when tau-related pathology disrupts large-scale functional systems involved in emotional salience, behavioral switching, and socioemotional regulation. This framework is consistent with the broader resting-state neuroimaging literature, demonstrating that AD preferentially may affect the default mode, salience, and limbic networks early in the disease course [29]. These networks are critically involved not only in memory and self-referential processing, but also in affective appraisal, motivation, and behavioral control. Collectively, these findings support a systems model in which MBI might represent the behavioral expression of selective network vulnerability within the AD continuum, linking regional tau pathology with NPS changes.

Importantly, emerging data suggest that different MBI domains may reflect distinct but overlapping pathogenic processes. In this regard, the MBI domain of psychotic manifestations may track tau-related change more closely, whereas decreased motivation and impulse dyscontrol may index broader metabolic and systems-level dysregulation [26]. Specifically, abnormal perception was associated with steeper increases in plasma pTau181 as mentioned above, whereas decreased motivation, and impulse dyscontrol were associated with homocysteine or insulin dysregulation [26]. Likewise, apathy might be particularly relevant in relation to tau-linked AD biomarker burden [30]. In particular, in older adults without dementia, the MBI domain of apathy was associated cross-sectionally and longitudinally with higher CSF p-tau181/Aβ42 and t-tau/Aβ42 ratios, and exploratory analyses also linked apathy with higher p-tau181 over a years-long follow-up [30]. Collectively, these findings support the view that MBI might be a biologically heterogeneous construct in which individual domains may reflect partially distinct—yet intersecting—molecular- and network-level mechanisms, reinforcing the value of domain-informed behavioral phenotyping for linking emergent NPSs to underlying AD pathophysiology.

Overall, MBI might represent a marker of higher amyloid burden, early tau accumulation, plasma p-tau181 elevation, and greater risk of clinical progression in AD [5,6]. This makes MBI particularly relevant in the AD continuum, where it may help identify patients with biologically active disease at a stage when behavioral change is already detectable but overt dementia has not yet developed.

### 4.2. MBI in Frontotemporal Spectrum Diseases Associated with FTLD-Tau Pathology

The behavioral variant FTD (bvFTD) is one of the most common clinical presentations of frontotemporal lobar degeneration (FTLD), representing the clearest illustration of the MBI construct in a non-AD context. Unlike AD, where memory loss typically dominates early presentations, bvFTD often begins with profound changes in behavior, personality, and social conduct that progressively worsen over time. These behaviors often emerge before measurable cognitive impairment, providing a strong rationale for considering MBI-like features as early indicators of bvFTD. This framework is particularly relevant in FTLD-tau, including Pick’s disease, the prototypical sporadic 3R tauopathy classically associated with early behavioral and socioemotional change. Notably, the five MBI domains map closely onto the core behavioral symptom clusters embedded in the FTDC criteria for possible bvFTD (disinhibition, apathy/inertia, loss of sympathy/empathy, perseverative/compulsive behaviors, hyperorality and dietary changes), supporting MBI as a structured syndromic lens for the earliest bvFTD phenotype [31].

Importantly, Taragano’s earlier work on late-life emergent behavioral changes without dementia helped motivate the later MBI framework [32,33,34]. According to Taragano and colleagues, MBI could be proposed to consist of four main features: (a) persistent behavioral alterations and mild psychiatric symptoms, particularly disinhibition; (b) absence of serious cognitive complaints; (c) normal daily functioning; (d) absence of dementia. In an early prospective cohort study by Taragano and colleagues, individuals with MBI showed a high risk of progression to dementia, including FTD and AD [32]. In another longitudinal cohort of individuals presenting with MBI, conversion occurred predominantly to bvFTD, with fewer AD converters [35]. Baseline executive dysfunction, severe impairment in theory of mind, and increased frontal atrophy were associated with higher conversion risk [35]. Furthermore, MBI has been associated with a higher risk of incident dementia, compared to the risk observed in a primary psychiatric comparison group, supporting the value of MBI in recognizing early, prodromal stages of FTD [36]. These findings support the view that incorporating social–cognitive testing and targeted frontal MRI readouts into MBI assessments may improve prognostic enrichment and reduce misclassification with primary psychiatric presentations.

Currently, the NPI, the NPI-Q and the Frontal Behavioral Inventory (FBI) are among the most widely used tools for evaluating neuropsychiatric symptoms in bvFTD [37,38]. However, the MBI and MBI-C are attracting increasing interest in assessing behavioral changes in patients with suspected bvFTD, especially at the early stages before the onset of prominent dementia [39]. Compared with the NPI or NPI-Q, which are typically anchored to shorter symptom windows and dementia populations, the MBI-C enforces persistence (≥6 months) and late-life onset, which may improve specificity for neurodegeneration-related behavioral change in bvFTD spectrum presentations [1]. One of the first cases of the application of the MBI-C in a patient with MBI who eventually developed bvFTD was published in 2018, where the MBI-C demonstrated better ability in identifying impulse dyscontrol symptoms compared to the NPI-Q [40].

The first study that investigated the application of the MBI-C in patients with bvFTD was by Cui and colleagues in 2023 [39]. In this Chinese cohort, including 52 patients with bvFTD and 82 healthy controls, MBI-C was greater than 0 in all patients with bvFTD and in about 40% of healthy controls [39]. The optimal cutoff point of the MBI-C for discriminating patients with bvFTD from healthy controls was 5.5, with 100% sensitivity and 83% specificity [39]. On the other hand, the NPI-Q for this discrimination had lower sensitivity and specificity measures compared to the MBI-C [39], suggesting that the MBI-C might be a more sensitive and specific tool for bvFTD compared to the NPI-Q. The optimal cutoff point of the Chinese version of the MBI-C in identifying AD dementia was 6.5 [41]. In accordance, a recent study indicated that the optimal cutoff point for distinguishing MCI due to AD from healthy controls in the Greek population was 9.5 [21]. A plausible explanation for the lower optimal MBI-C cutoff in bvFTD compared with AD is that behavioral and personality changes are the core, early features of bvFTD, so even a modest symptom burden might more reliably distinguish patients with bvFTD from controls without bvFTD. In contrast, NPSs in AD dementia might be more heterogeneous in profile and timing, often emerging alongside broader cognitive decline, which can shift the threshold needed to maximize discrimination. Moreover, a substantial proportion of healthy controls may score above zero on the MBI-C, so a somewhat higher cutoff in AD studies may be required to limit false positives. Furthermore, Dodich et al. showed that patients with bvFTD exhibited more prominent and more specific theory-of-mind impairment compared to AD [42], suggesting that early social inappropriateness in MBI might reflect disease-specific disruption of socioemotional inference rather than generalized dementia severity. Hence, in bvFTD, even modest behavioral changes might be diagnostically informative at an earlier point; meanwhile, in AD, a higher overall symptom burden may be needed to optimize discrimination.

In the study by Cui and colleagues, in patients with bvFTD, apathy was the most common MBI domain, followed by impulse dyscontrol, affective dysregulation, social inappropriateness and psychosis [39]. In subgroup analyses, impulse dyscontrol and apathy were the most prevalent MBI domains in mild and moderate-to-severe FTD, respectively [39]. The relative frequency of the MBI domains in bvFTD is broadly consistent with the clinical prominence of apathy, disinhibition, loss of empathy, and compulsive or perseverative behaviors in bvFTD as reflected in FTDC-based literature [31].

Regarding the neuroimaging correlates, apathy in bvFTD has been associated with atrophy in midline prefrontal regions, including areas of the orbitofrontal cortex, anterior/dorsal cingulate cortex [43], as well as fractional anisotropy changes in inferior fronto-occipital fasciculus and forceps minor [44]. Disinhibition has been linked to altered fiber integrity in the superior longitudinal fasciculus [44]. Moreover, Whitwell and colleagues demonstrated substantial neuroanatomical heterogeneity in bvFTD, identifying four distinct MRI-based atrophy subtypes (frontal-dominant, frontotemporal, temporal-dominant, and temporofrontoparietal). Notably, these subtypes differed on cognitive measures, including episodic memory, executive function, and confrontation naming, but showed comparable behavioral severity as being indexed by the NPI, suggesting that a similar overall NPS burden may accompany divergent regional patterns of neurodegeneration [45]. This anatomical heterogeneity supports domain-based behavioral phenotyping as a complementary approach to syndrome labels, because similar overall NPS burden may arise from divergent network-level degeneration patterns. However, it would be interesting for future studies to investigate the underlying neuroanatomical and functional correlations of MBI domains in bvFTD, especially at the earliest stages, where the sensitivity of MBI-C might be higher in detecting subtle cases.

In bvFTD, impairment in social cognition is a core early feature that overlaps substantially with the MBI domain of social inappropriateness. Rankin and colleagues showed that patients with FTLD demonstrate distinct disturbances in empathy [46,47], supporting the view that empathy loss reflects primary disease-related disruption of socioemotional processing. Consistent with this, the international consensus recommendations on differentiating bvFTD from primary psychiatric disorders emphasize that at least one formal social cognition test should be included in the standard assessment [47], as social–cognitive deficits might be considered particularly informative when behavioral symptoms dominate and cognitive screening may still be relatively preserved. Given that deficits in empathy and social cognition often precede overt cognitive impairment, the NIC-FTD consensus recommends incorporating at least one formal social cognition measure into the routine assessment when bvFTD is suspected, an approach that aligns directly with the MBI domain of social inappropriateness and may reduce psychiatric misdiagnosis. Together, these data reinforce the clinical value of systematically capturing MBI “social inappropriateness” features in the suspected early stages of bvFTD, both to support earlier detection and to reduce misdiagnosis as a primary psychiatric condition.

In the study by Cui and colleagues, all MBI domains, with the exception of psychotic features, were more common in patients with bvFTD compared to healthy controls. Interestingly, the prevalence of psychosis was similar between people with mild bvFTD and healthy controls, whereas it was higher in the subgroup of people with moderate-to-severe bvFTD [39]. This pattern suggests that most MBI domains are sensitive to early bvFTD, whereas psychotic symptoms might usually emerge later in the disease course, and therefore may be less useful for early-stage discrimination. Importantly, no significant associations were observed between the MBI-C and cognitive scales in the study by Cui and colleagues, including both global (MMSE, MoCA) and domain-specific tests (Auditory Verbal Learning Test (AVLT) learning, AVLT Delayed recall, AVLT Cued recall, Trail Making Test Part A (TMT-A), Trail Making Test Part B (TMT-B), and Boston Naming Test (BNT)) [39]. No correlation was also found between MBI-C and the sum of Clinical Dementia Rating (CDR) [39]. In accordance, there are several reports suggesting that NPSs in FTD, including apathy, are not directly correlated to cognitive performance, including executive function [43]. On the other hand, the MBI-C was at least weakly correlated with ADL score, supporting the consideration that the daily functioning of patients with bvFTD is influenced at least to some extent by NPS [39], and that the MBI, by definition, is accompanied by at least minimum impaired daily functioning [1].

Concerning genetic FTSD, using Genetic FTD Initiative (GENFI) data, Tavares et al. examined symptomatic mutation carriers in *MAPT*, *GRN* and *C9orf72*, as well as their at-risk relatives to define the earliest clinical features of genetic FTLD [48]. In symptomatic individuals, the most frequently endorsed initial symptoms were apathy and disinhibition, followed by cognitive deficits including memory impairment and decreased fluency [48]. In the preclinical stage, *MAPT* carriers reported more mood- and sleep-related symptoms, *C9orf72* carriers demonstrated a slightly greater degree of abnormal behaviors, while *GRN* carriers showed fewer mood symptoms compared to non-carriers [48]. Overall, the findings highlight that early behavioral symptoms, particularly the MBI-related domains of decreased motivation and impulse dyscontrol, might be common first manifestations in genetic FTD cases, and may serve as clinically meaningful endpoints for prodromal genetic FTD studies. On the other hand, affective dysregulation domain reflected by mood symptoms might be early, underrecognized signs of prodromal FTD especially in *MAPT* mutation carriers. In accordance, another GENFI cohort study indicated that the frequency and severity of NPSs differed by genotype (*MAPT*, *GRN*, *C9orf72*) in FTD, showing distinct trajectories across disease stages [49]. *MAPT* carriers exhibited the highest frequency and severity of several core behavioral features, particularly disinhibition and compulsive behaviors, compared with *C9orf72* and *GRN* carriers [49]. Anxiety and depression were most prominent in *GRN* and *MAPT* carriers, whereas hallucinations, including auditory and visual types, were most frequent in *C9orf72* carriers [49]. Importantly, psychosis spectrum phenomena appear to carry staging value beyond standard clinical staging scales, supporting their systematic assessment especially in prodromal genetic FTSD [50]. Across the disease course, most symptoms increased in early–intermediate stages and then plateaued, highlighting genotype-specific behavioral signatures that are relevant for phenotyping and trial design. In particular, the disproportionally higher frequency of the MBI domain of psychosis in *C9orf72* FTD carriers supports this feature as a potential genotype-enriched prodromal signal rather than a nonspecific late complication. This evidence aligns with the findings by Cui et al. described above, where psychosis was a generally rare manifestation in bvFTD [39]. Thus, systematic capture of psychotic phenomena within an MBI framework may improve early phenotyping and aid suspicion of *C9orf72*-associated FTSD, particularly when prominent psychosis accompanies subtle behavioral change.

In another study based on a large GENFI cohort of presymptomatic *MAPT*, *GRN*, and *C9orf72* mutation carriers, followed annually with MRI for two years, apathy increased over time in carriers but not in non-carrier relatives [51]. Importantly, baseline apathy predicted subsequent cognitive decline over the follow-up period, whereas baseline cognition did not predict worsening apathy [51]. Progression of apathy was linked to lower baseline gray-matter volume in frontal and cingulate regions, suggesting a neuroanatomical substrate in motivational control networks [51]. Together, these data position apathy in the context of decreased motivation MBI domain as a potential prodromal neurobehavioral marker in presymptomatic genetic FTD that anticipates subclinical cognitive deterioration.

In summary, current evidence supports MBI as a promising framework for identifying the earliest NPSs of FTSD. By capturing persistent late-life behavioral change across key domains, MBI may improve early recognition, refine phenotyping, and strengthen links between NPSs and underlying neurodegenerative pathology.

### 4.3. MBI in Four Repeat (4R)-Tauopathies

4R-tauopathies constitute a subgroup of primary tauopathies characterized by the abnormal aggregation of tau isoforms containing four microtubule-binding repeats. Under physiological conditions, alternative splicing of the *MAPT* gene generates tau isoforms with either 3R or 4R domains. In 4R-tauopathies, this balance is disrupted, leading to preferential accumulation of 4R tau in neurons and glial cells [52]. The main clinicopathological entities in this group include PSP and CBD, although 4R tau pathology may also underly other phenotypes within the FTLD spectrum [52]. Unlike AD, which contains mixed 3R/4R tau and is typically associated with concomitant amyloid pathology, primary 4R tauopathies are defined by distinct molecular, cellular, and anatomical patterns of tau deposition, with prominent involvement of subcortical, brainstem, and frontostriatal networks [53]. These disorders are therefore of particular interest in studies of MBI, because their regional tau burden can be closely linked to syndromes of apathy, dysexecutive dysfunction, behavioral change, and impaired social–emotional regulation.

Dedicated studies examining MBI as a formal syndrome in primary 4R-tauopathies remain very limited, and this represents an important gap in the literature. At present, most relevant evidence comes indirectly from studies of neuropsychiatric and behavioral symptoms in PSP and corticobasal syndrome (CBS) or CBD, rather than from MBI-C-based or ISTAART-AA-anchored investigations.

The available evidence suggests that behavioral changes are clinically relevant early in 4R-taupathies and may be particularly informative in relation to underlying 4R-tau network degeneration. In PSP, apathy is one of the most consistent neuropsychiatric features, with a weighted mean prevalence of approximately 60% across studies, and it may be identifiable even in relatively early disease stages, where it can help distinguish PSP phenotypes from PD [54].

In newly diagnosed PSP and CBS cohorts, apathy, aspontaneity, depression, irritability, and language-behavioral changes have all been described, with apathy more frequent in PSP than CBS (approximately 58% versus 34%) in one study [55]. Moreover, apathy and impulsivity frequently co-occur in PSP, supporting the idea that these syndromes might reflect a disruption of shared frontostriatal and subcortical regulatory systems [56]. This possible interpretation is biologically plausible, as behavioral severity in PSP has been linked to volume loss in the lateral posterior frontal cortex, a region within the frontostriatal networks; meanwhile, apathy specifically has been associated additionally with putaminal atrophy [57], potentially reflecting broader subcortical degeneration.

Complementary 7T MRI data further implicate locus coeruleus (LC) degeneration in apathy and cognition in PSP, suggesting a potential tau-mediated noradrenergic dysfunction in PSP [58]. Interestingly, in comparison to AD and healthy ageing where higher levels of neuronal loss have been shown in the rostral LC, the caudal subregion was mostly affected in PSP in this study [58]. It has been hypothesized that the caudal subregion might be particularly vulnerable to potential environmental toxic stimuli via the CSF due to the proximity to the fourth ventricle [58]. Furthermore, the caudal subregions of the LC receives vagal nerve projections through the solitary tract nucleus, which might exert higher vulnerability to misfolded proteins such as tau protein, transmitted from the periphery [58]. These observations suggest that early NPSs in PSP might arise not only from frontostriatal degeneration, but also from involvement of brainstem neuromodulatory systems, with the LC representing a plausible link between 4R-tau pathology and the emergence of apathy and cognitive dysfunction.

In CBS/CBD, early behavioral presentations appear somewhat less stereotyped, but frontal behavioral symptoms, depression, compulsive features, and irritability are well recognized [59], and early frontal-type behavioral changes may even help predict underlying CBD rather than AD pathology in patients presenting with CBS [60].

Overall, although it would be premature to claim an established MBI phenotype for 4R-tauopathies, the available evidence supports the hypothesis that later-life-emergent apathy, impulse dyscontrol, aspontaneity, and related frontal–subcortical behavioral syndromes may represent clinically meaningful prodromal or early manifestations of PSP and CBD, warranting future studies that apply formal MBI criteria and domain-based phenotyping in these disorders.

## 5. Potential Underlying Molecular Mechanisms of MBI in Tauopathies

### 5.1. Tauopathies and Network-Specific Behavioral Disruption in AD

In AD, abnormal tau inclusions seem to follow a spreading pattern in a predictable manner in the brain, including six stages (Braak I-VI) [61]. According to these early postmortem observations, as well as PET evidence using tau ligands suggest that the NFTs in AD appear in a relatively stereotypical spatiotemporal pattern, starting from the brainstem and the trans-entorhinal cortex and spreading progressively to synaptically connected neuronal circuits [62]. This hierarchical progression has been interpreted within the framework of trans-neuronal tau propagation, whereby misfolded tau species act as “seeds” that template the aggregation of native tau in anatomically connected neurons. In vitro and in vivo evidence has demonstrated that pathological tau can be released from affected neurons, internalized by neighboring cells, and subsequently induce intracellular tau aggregation, thereby enabling propagation along functional neuroanatomical pathways [63,64].

Network-based analyses of tau PET imaging indicate that pathological tau deposition is strongly associated with large-scale functional connectivity patterns, with the most prominent accumulation occurring in key nodes of the default mode network, including the posterior cingulate cortex, precuneus, angular gyrus, and lateral temporal cortex, with broader anterior neocortical involvement in more advanced stages as disease progresses [65]. These findings support the assumption that tau pathology might spread preferentially along functionally connected neural systems rather than randomly across cortical regions, consistent with connectome-based models of neurodegeneration [66]. In particular, default mode network hubs appear especially vulnerable to tau accumulation and possibly act as convergence zones for pathological propagation across different clinical phenotypes of AD [62,65]. Importantly, salience-related and limbic regions, particularly the anterior cingulate and insula, together with orbitofrontal areas involved in socioemotional valuation, play key roles in emotional salience processing, social cognition, and motivational regulation [67,68].

Early tau-related disruption of these circuits may contribute to the emergence of NPSs in the context of MBI. In this regard, it can be hypothesized that the neurodegeneration within the salience network, particularly involving the anterior insula and anterior cingulate cortex, might impair the integration of emotional and interoceptive signals [67], thereby contributing to the MBI domains of affective dysregulation and impulse dyscontrol. Similarly, involvement of default mode and limbic network hubs, including the medial temporal lobe, orbitofrontal cortex, and posterior cingulate cortex, may interfere with social cognition, self-referential processing, and motivational behavior, potentially manifesting clinically as apathy, reduced empathy, and altered social behavior [68]; these are features that overlap mainly with the decreased motivation and social inappropriateness MBI domains.

At the cellular level, the tau-mediated disruption of axonal transport can impair the trafficking of essential elements along axons, including mitochondria, and synaptic vesicles, thereby affecting synaptic survival and diverse cellular functions [22]. Furthermore, the tau-mediated impairment of mitochondrial transport along axons and dendrites can also result in cellular energy deficits and oxidative vulnerability at synaptic terminals, thereby enhancing neuronal vulnerability [22]. In experimental tauopathy in vivo models, impaired axonal transport has been associated with microstructural white-matter injury, including reduced content of myelin [69], further supporting a mechanistic link between tau-mediated cytoskeletal dysfunction and network disconnection syndromes.

At the synaptic level, tau mislocalization to dendritic spines disrupts glutamatergic transmission, alters NMDA receptor trafficking, and ultimately leads to synaptic loss [22]. Such synaptic dysfunction is considered one of the earliest cellular events in tau-mediated neurodegeneration and may precede overt neuronal loss [11,22]. In particular, it has been recently shown that soluble, oligomeric tau species can exert synaptotoxic effects [22], thereby representing possible early drivers of circuit inefficiency. Importantly, post-mortem evidence has shown that oligomeric forms of tau exist in both pre- and post-synaptic terminals in the cortex of patients with AD, even in regions without prominent deposition of fibrillar tau protein [70]. Furthermore, increased levels of oligomeric tau forms have been observed in synaptic terminals disproportionally to phosphorylated and misfolded tau forms [70]. Collectively, these findings suggest that the abnormal accumulation of oligomeric tau in the synapses occurs early during the tau-related neurodegenerative process, and that the progression of tau proteinopathy in the brain might be mediated by its trans-synaptic spreading.

From a clinical perspective, this network-selective vulnerability provides a compelling framework that could link tau propagation with the emergence of NPSs in the context of MBI. Early tau deposition within limbic-salience circuits may impair emotional salience attribution and socioemotional processing, potentially manifesting as subtle behavioral changes such as apathy or irritability before cognitive decline. As tau pathology spreads from medial temporal regions to medial prefrontal and orbitofrontal hubs, it can be hypothesized that the progressive disruption of frontostriatal and frontolimbic circuits might further amplify these behavioral disturbances, contributing to the longitudinal evolution of MBI domains across the neurodegenerative continuum. Thus, it can be proposed that the prion-like propagation model of tau pathology might at least partially underlie the early NPSs in the context of MBI in AD via network-level dysfunction.

### 5.2. MBI as a Clinical Manifestation of Early Brainstem Tau Pathology in AD

Notably, abnormal alterations in tau protein, including phosphorylation at certain epitopes, have been observed in the LC in the brainstem in a substantial proportion of young individuals in their 20s, many years before the emergence of amyloid-beta pathology [71]. In this large postmortem study, the transentorhinal region was the first cortical area affected by tau pathology, where the transentorhinal pyramidal cells demonstrated pretangles, which were progressively converted into argyrophilic NFTs according to Stages I to VI [71]. Importantly, amyloid-beta plaques appeared in cortical regions only after the initial development of brainstem tauopathy, suggesting that tau-related neurodegenerative processes may precede and potentially facilitate later amyloid pathology [71].

The LC plays a central neuromodulatory role in the brain through its widespread noradrenergic projections, which regulate arousal, emotional salience processing, attention, stress responsiveness, and cognitive flexibility [72]. LC neurons project extensively to limbic and cortical regions, including the hippocampus, amygdala, anterior cingulate cortex, and prefrontal cortex, thereby modulating neural circuits that are critically involved in emotional regulation, motivational behavior, and social cognition [72]. Degeneration or dysfunction of LC neurons, therefore, might potentially lead to early disturbances in noradrenergic signaling that alter the functional integrity of these frontolimbic and salience networks. It can be hypothesized that the early tau pathology in LC in AD, observed in several studies [73,74], might disrupt noradrenergic modulation long before overt neuronal loss and overt cognitive decline, potentially subtly affecting emotional reactivity, stress processing, behavioral inhibition, and goal-directed motivation, processes that are central to the MBI-related symptoms.

In addition to the LC, the dorsal raphe nucleus (DRN), the principal serotonergic nucleus of the brainstem, has also been implicated as an early site of tau pathology in AD. Neuropathological evidence shows that phospho-tau changes may appear in the supratrochlear subnucleus of the DRN even before the involvement of the transentorhinal cortex [75], further supporting the hypothesis that tauopathy may begin in brainstem monoaminergic nuclei before spreading to limbic and cortical regions. Importantly, the DRN contains the largest population of serotonergic (5-hydroxytryptamine, 5-HT) neurons in the brain, projecting extensively to limbic, cortical, and subcortical regions (including the hippocampus, amygdala, anterior cingulate cortex, hypothalamus, and prefrontal cortex) [76]. These serotonergic projections play key roles in mood regulation, stress reactivity, reward processing, sleep–wake regulation, and emotional behavior, functions that are frequently altered during the prodromal phases of neurodegenerative disease [76]. Tau pathology has been observed in the DRN of young individuals even in their 20s, with its prevalence being comparable to that of LC [76]. In contrast, other pathologies, such as beta-amyloid plaques, a-synucleinopathy or TDP-43 proteinopathy appeared less frequently in this study [76]. Interestingly, mice overexpressing the human P301L-tau in their DRN have been shown hyperactivity and depressive-like manifestations, with their spatial memory remaining intact [76]. In this study, these behavioral changes were accompanied with hyperexcitable 5-HT neurons, higher astrocytic density, as well as increased expression of interleukin (IL)-1a and Frk, suggesting upregulated neuroinflammatory pathways [76]. Furthermore, tau pathology was identified in the neuraxonal processes in the amygdala, thalamus, hypothalamus and caudate putamen, frequently co-localizing with the serotonin reuptake transporter (SERT) [76]. Collectively, these results suggest that the DRN might be at least one of the earliest regions affected by tau pathology in AD, possibly related to early behavioral changes, which could progressively spread into other brain areas in an anterograde manner [76].

Taken together with the early involvement of the noradrenergic LC, these findings suggest that tau-mediated dysfunction of monoaminergic neuromodulatory systems may represent one of the earliest neurobiological events in AD and related tauopathies. Because the LC and DRN collectively regulate large-scale limbic and salience network activity through noradrenergic and serotonergic projections, early degeneration within these nuclei could produce disturbances in emotional regulation, motivational drive, and behavioral inhibition. In this regard, MBI might reflect the earliest detectable clinical expression of a neurodegenerative process that initially affects neuromodulatory brainstem nuclei before spreading to cortical association networks.

### 5.3. Potential Mechanistic Links Between MBI and Non-AD Tauopathies

Hyperphosphorylation has been shown to modulate the propagation capacity of tau protein, thereby affecting the morphology of downstream lesions in vivo [77]. Abnormal phosphorylation of tau reduces its affinity for microtubules and promotes its detachment from axonal cytoskeletal structures, resulting in destabilization of microtubules and impaired axonal transport [64]. These molecular alterations favor the formation of misfolded tau species that can act as seeding-competent aggregates, while hyperphosphorylated tau seeds exhibit enhanced capacity for intercellular transmission. In addition to altering aggregation kinetics, phosphorylation-dependent conformational changes may generate distinct “tau strains,” each characterized by unique biochemical and structural properties that influence the regional vulnerability and clinical phenotype of tauopathies [78,79]. Such strain diversity has been proposed to contribute to the heterogeneity across tau-related disorders, including AD, PSP, CBD, and Pick’s disease [78,79]. These disease-specific tau conformations might preferentially propagate along specific neural circuits, thereby producing distinct patterns of network degeneration and behavioral manifestations.

Pick’s disease, a primary 3R-tauopathy, is neuropathologically characterized by the presence of Pick bodies, constituting spherical neuronal inclusions composed predominantly of hyperphosphorylated 3R tau isoforms, along with neuronal loss and gliosis, particularly within the frontal and anterior temporal cortices [80]. These regions include areas implicated in the salience and social cognition networks, including the orbitofrontal cortex, anterior cingulate cortex, and temporal poles. Individuals with Pick’s disease frequently exhibit early behavioral manifestations such as disinhibition, apathy, compulsive behaviors, and impaired empathy [80]. At the molecular level, tau aggregation within frontotemporal neurons disrupts microtubule dynamics, axonal transport, and synaptic signaling, ultimately producing network-level dysfunction within circuits responsible for socioemotional processing and behavioral regulation [80]. It can be proposed that, within the MBI framework, these early disturbances may manifest clinically as impulse dyscontrol, social inappropriateness, and affective dysregulation, providing a potential behavioral correlate of early tau-mediated circuit dysfunction in this tauopathy.

Large genetic FTLD cohorts have demonstrated that carriers of some *MAPT* variants may exhibit a particularly high frequency and severity of core behavioral features, particularly disinhibition and compulsive behaviors [49]. These findings suggest a potential genotype-related vulnerability of orbitofrontal–striatal and anterior temporal regions, which might be phenotypically related to the MBI impulse dyscontrol and affective dysregulation domains. Importantly, the clinical phenotypes of *MAPT* carriers can be heterogeneous, including “AD-like” presentations in some variants. However, NPSs such as disinhibition and aggressiveness remain common across the different phenotypic expressions, reinforcing the value of an MBI framework to capture potential “trans-syndromic” behavioral changes [81]. This genotype-phenotype correlation supports the hypothesis that tau-mediated synaptic and axonal dysfunction within orbitofrontal–striatal and anterior temporal networks might possibly generate MBI-like presentations, particularly within impulse dyscontrol and in some cases affective dysregulation domains.

PSP, a prototypical 4R-tauopathy, is characterized by tau accumulation mainly in subcortical and brainstem structures, including the midbrain, basal ganglia, thalamus, and cerebellar nuclei, along with involvement of frontal cortical regions [54,56,82]. Although PSP is traditionally considered a movement disorder, NPSs are common and may emerge early in the disease course, with apathy being the most prevalent behavioral feature [54,56,82]. Neuroimaging and neuropathological studies suggest that NPSs in PSP are associated with degeneration of frontostriatal circuits and the anterior cingulate cortex, which play central roles in motivational and goal-directed behavior [54,56,82]. In this context, tau-mediated disruption of frontostriatal and cingulate networks may produce early MBI-related symptoms within the decreased motivation domain, while additional involvement of orbitofrontal circuits may contribute to behavioral inflexibility and impulse dyscontrol.

CBD, another 4R-tauopathy, is characterized by widespread tau accumulation in both cortical and subcortical regions, including the frontal cortex, parietal cortex, basal ganglia, and white matter [83]. Although CBD often presents with asymmetric motor symptoms, cognitive impairment and NPSs, including apathy, depression and irritability, are integral and often early clinical components [83,84]. At the molecular level, tau pathology in CBD involves neuronal and glial inclusions, including astrocytic plaques and coiled bodies, suggesting that glial dysfunction and neuroinflammation might possibly contribute to network instability [83,84]. Degeneration within frontoparietal and frontostriatal circuits, which serve executive control, emotional regulation, and social cognition, might therefore produce behavioral phenotypes overlapping with MBI domains, particularly decreased motivation, affective dysregulation, and impulse dyscontrol.

Collectively, the clinical and molecular characteristics of Pick’s disease, PSP, and CBD further support the notion that tauopathies may preferentially disrupt large-scale socioemotional and executive networks long before widespread neuronal loss occurs. Since these circuits are critically involved in motivation, emotional processing, and social behavior, MBI can be conceptualized as a behavioral expression of early network vulnerability across multiple tauopathies before the onset of overt dementia or advanced motor syndromes.

### 5.4. Synaptic Dysfunction as a Possible Underlying Mechanism of MBI in Tauopathies

**Figure 1:**
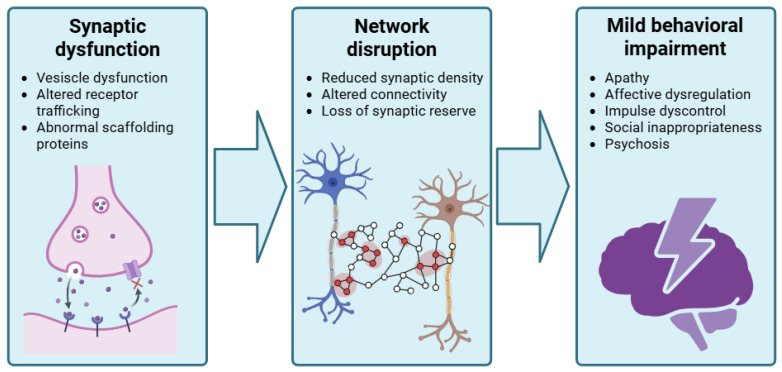
**Synaptic dysfunction as neurobiological underpinning for mild behavioral impairment.** Early alterations at the synapse, including changes in vesicle proteins, receptor trafficking, and scaffolding elements, affect network connectivity. Clinically, these changes may manifest as behavioral symptoms characteristic of MBI, before overt cognitive impairment. Created with BioRender.com. Villa, C. (2026) https://BioRender.com/4llgthn, accessed on 30 March 2026.

A shared mechanism among neurodegenerative diseases is synaptic disruption, representing an early event during the pathophysiological process (Figure 1). In this regard, MBI can be hypothesized to reflect an “intermediate” phenotype of loss of “synaptic reserve”, where cognitive decline impairment is not yet prominent.

Early molecular changes in the synapses, including vesicle proteins, scaffolding proteins and receptor trafficking machinery, might affect network dynamics even before prominent synapse elimination or neuronal loss becomes evident. Neuroimaging evidence including PET studies with synaptic vesicle glycoprotein 2A (SV2A), a presynaptic marker, has indicated reduced synaptic density in neocortical and medial temporal regions in early AD, to a greater extent compared to gray matter volume loss [85]. Similarly, in bvFTD, PET imaging has shown reduced synaptic density across medial and dorsolateral frontal cortex, anterior cingulate cortex, insula and medial temporal regions, more extensive than expected from grey matter atrophy [86]. PET studies with SV2A tracers in PSP and CBD have demonstrated widespread reductions in synaptic density in frontal, limbic and subcortical regions, often reaching 20-50% decreases in regions with minimal cortical atrophy [87]. Moreover, longitudinal evidence shows that synaptic density declines progressively over time and correlates with clinical deterioration in PSP and CBD [87], highlighting synaptic loss as a key driver of network failure in primary tauopathies.

### 5.5. Neurotransmitter Imbalance, Tauopathies and MBI

Beyond synaptic dysfunction, disruption of major neurotransmitters may further contribute to the early behavioral phenotypes captured within the MBI construct. The cholinergic system, originating primarily from the basal forebrain nuclei, including the nucleus basalis of Meynert, plays a critical role in the regulation of behavior. Although cholinergic degeneration is classically associated with AD, emerging evidence suggests that cholinergic dysfunction may also occur in FTSD [88]. Reduced cortical cholinergic innervation and altered vesicular acetylcholine transporter (VAChT) binding has been demonstrated in frontotemporal regions in patients with bvFTD [88], suggesting that impaired cholinergic modulation of prefrontal networks may contribute to deficits in executive control and social cognition.

The mesocorticolimbic dopamine system, projecting from the ventral tegmental area to the prefrontal cortex and striatum, plays a central role in reward processing, motivation, and goal-directed behavior [89]. Neuroimaging studies using dopamine transporter (DAT) SPECT and PET ligands have demonstrated reduced striatal DAT binding and broader dopaminergic dysfunction in patients with bvFTD [90]. Post-mortem studies in FTLD have demonstrated reduced cortical serotonin receptor binding, particularly involving the 5-HT1A and 5-HT2A receptor subtypes [91]. Similarly, degeneration of the noradrenergic LC can impair attentional regulation, stress responsivity, and emotional salience processing, as mentioned above. Taken together, these findings suggest that early disruptions across dopaminergic, serotonergic, and noradrenergic systems might converge to possibly destabilize frontolimbic and frontostriatal networks that regulate motivation, emotional reactivity, and behavioral inhibition, thereby providing a neurochemical substrate through which MBI may emerge as one of the earliest clinical manifestations of neurodegenerative disease.

### 5.6. Neuroinflammation and Glial Contributions in Tauopathies: Insights for MBI

Neuroinflammation is increasingly recognized as an active component of tau-mediated neurodegeneration. Both microglia and astrocytes appear to participate in a bidirectional process in which pathological tau induces glial activation, while reactive glia in turn amplify tau accumulation, and synaptic dysfunction [92].

In AD, human and experimental data suggest that microglial activation is closely coupled to tau pathology and may facilitate its spread. In particular, microglia can internalize soluble and aggregated tau, but when degradation is inefficient, they can re-release seed-competent tau through extracellular vesicles, thereby promoting non-synaptic propagation [92]. In tauopathy models, depletion of microglia or inhibition of microglial exosome synthesis has been shown to reduce phosphorylated tau burden and limit tau spread between connected regions [92]. Mechanistically, several inflammatory pathways have been implicated, including triggering receptor expressed on myeloid cells 2 (TREM2)-dependent signaling, nuclear factor kappa-light-chain-enhancer of activated B cells (NF-κB) activation, and the NLRP3/ASC inflammasome, all of which can modulate tau seeding, clearance, and neurotoxicity [93,94,95]. Notably, complement signaling appears especially important, as in PS19 tauopathy mice, deletion of C3aR attenuated tau pathology, has been associated with reduced neuroinflammation, improved synaptic integrity, and reversion of a reactive glial transcriptional program linked to neurotoxic astrocytes [96].

Astrocytes also appear to play a central role in AD. Astrocyte reactivity marked by GFAP is linked to synaptic dysfunction in vivo. In a CSF biomarker study, higher CSF GFAP was associated with both presynaptic and postsynaptic dysfunction markers, whereas sTREM2 showed a more Aβ-dependent association [97]. Importantly, CSF pTau181 was demonstrated to mediate the relationship between glial reactivity and synaptic dysfunction [97]. Post-mortem evidence further supports an active glial role in early synapse elimination. In a post-mortem study, individuals with early dementia and Braak III–IV pathology showed a 43% reduction in presynaptic elements, 33% reduction in postsynaptic elements, and 38% reduction in mature colocalized synapses compared with controls [98]. In the same study, the proportion of mature synapses internalized by microglia was approximately 13% in dementia versus 3% in resilient individuals and 1% in controls, while astrocytic internalization was 17% versus 4% and 3%, respectively [98]. Collectively, these findings are highly relevant to MBI, because they suggest that glia-mediated synaptic injury can emerge at stages when overt neuronal loss is still limited, potentially producing early behavioral network dysfunction before prominent cognitive decline.

In PSP and CBD, in addition to neurons, tau accumulates also in glial cells, forming characteristic lesions such as tufted astrocytes in PSP and astrocytic plaques and oligodendroglial pathology in CBD [99,100]. Clinicopathologic studies indicate that the overall primary FTLD-tau burden in PSP and CBD reflects the combined burden of neuronal, astrocytic, and oligodendroglial tau pathology, and this total burden correlates with clinical deficits more strongly than copathologies [101]. In PSP, plasma GFAP has been shown to distinguish PSP from healthy controls and MSA-P, and it correlated with brainstem atrophy and regional tau accumulation, although neurofilament light (NfL) remained the stronger overall marker [102].

Recent work also suggests that astrocytes may actively shape tau handling in 4R tauopathies. Astrocytes preferentially take up 4R tau more readily than 3R tau in vitro, and this uptake is impaired under inflammatory or metabolic stress [103], providing a possible mechanism for the predominance of astrocytic tau lesions in PSP and CBD. In human PSP tissue and living human brain slice models challenged with PSP-derived tau, astrocytes showed increased synaptic engulfment and astrogliosis, whereas microglial activation was less prominent in frontal cortex [104]. Tau immunodepletion prevented tau-induced astrogliosis and astrocytic synaptic engulfment [104], suggesting that astrocytes may directly contribute to synapse loss in PSP. This observation is particularly relevant for MBI, because astrocyte-mediated synaptic pruning within frontal and cingulate regions might possibly contribute to early apathy, behavioral inflexibility, and socioemotional dysfunction in PSP spectrum disease.

In FTLD-tau more broadly, neuroinflammation also appears biologically relevant. Review and neuropathologic data indicate increased microglial and astrocytic activation in human FTLD-tau and tauopathy models [105], along with elevated inflammatory biomarkers and TSPO-PET signal in some cohorts [106]. Importantly, genetic loci relevant to innate immune signaling, including TREM2, have been linked to FTD-related disease biology, supporting the idea that glial dysregulation is not merely epiphenomenal [107,108]. Experimental work suggests that astrocyte-derived APOE4 may be especially deleterious in tauopathy, as selective removal of astrocytic APOE4 in a P301S model reduced tau-mediated neurodegeneration, decreased synaptic loss, and reduced microglial phagocytosis of synaptic elements [109].

**Figure 2:**
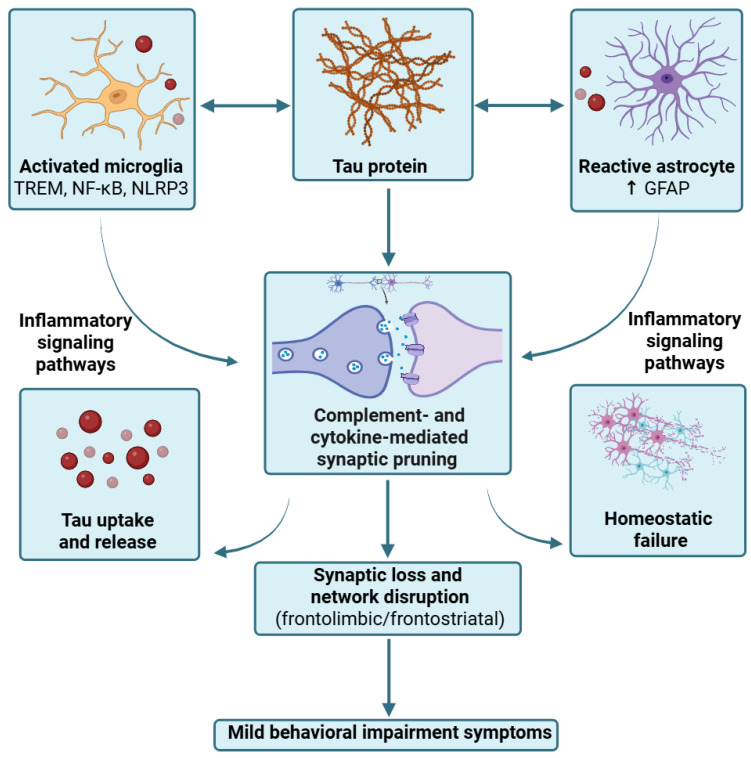
**Neuroinflammation as a pathway linking tau pathology to early behavioral symptoms via synaptic dysfunction.** Pathological tau accumulation activates microglia and astrocytes, establishing a bidirectional feed-forward loop that promotes tau aggregation and propagation. Microglia contribute to tau spread by taking up and releasing tau via extracellular vesicles, whereas inflammatory signaling pathways regulate tau toxicity and clearance. In parallel, synaptic pruning is driven by complement- and cytokine-mediated mechanisms, with both microglia and astrocytes actively engulfing pre- and postsynaptic elements. Astrocyte reactivity may also impair metabolic and homeostatic support for neuronal networks. These processes converge in synaptic loss and network disruption, particularly in frontolimbic and frontostriatal circuits, eventually leading to MBI-specific behavioral manifestations. Created with BioRender.com. Villa, C. (2026) https://BioRender.com/5rr28vy, accessed on 30 March 2026.

Taken together, these findings support a model in which glial responses might potentially contribute to MBI-relevant behavioral changes through at least three converging mechanisms: (i) amplification of tau propagation through microglial uptake and extracellular vesicle release; (ii) complement- and cytokine-driven synaptic pruning by microglia and astrocytes; (iii) astrocytic and microglial failure to maintain synaptic and network homeostasis in vulnerable frontolimbic and frontostriatal circuits. In this framework, neuroinflammation could potentially represent a mediator linking molecular tau pathology to the circuit-level dysfunction that may be clinically expressed as emergent MBI symptoms (Figure 2).

### 5.7. Integrative Network Perspective: From Molecular Correlates to MBI Domains

**Figure 3:**
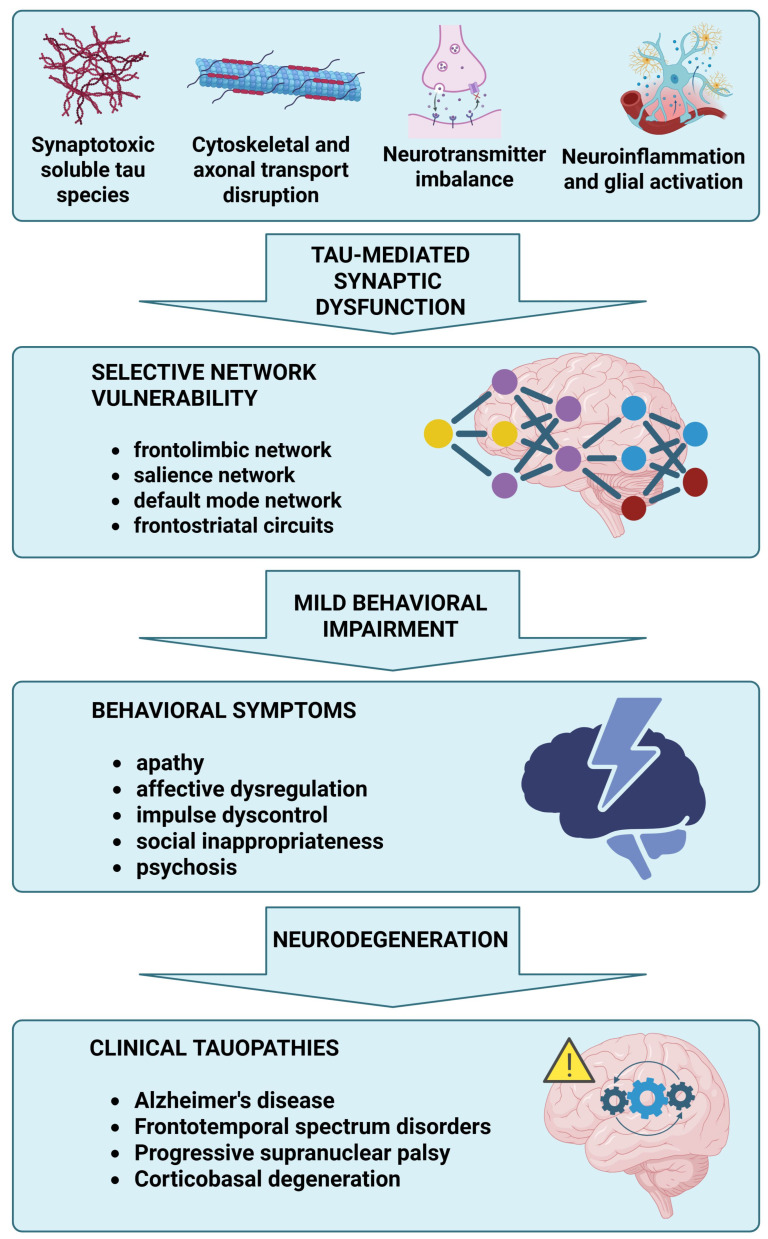
**From mild cognitive impairment to tauopathies: a mechanistic framework.** Pathological tau processes, including synaptotoxic soluble tau species, disrupted cytoskeletal and axonal transport, neurotransmitter imbalance, and neuroinflammation, all lead to early synaptic dysfunction. These changes target large-scale brain networks involved in behavioral control, such as the frontolimbic, salience, default mode, and frontostriatal circuits. Dysfunction in these systems might present clinically as mild behavioral impairment, characterized by persistent late-life behavioral alterations such as apathy, affective dysregulation, impulse dyscontrol, social inappropriateness, and psychosis. These symptoms may represent early signs of neurodegeneration, preceding the onset of overt clinical tauopathies such as Alzheimer’s disease, frontotemporal spectrum diseases, progressive supranuclear palsy, and corticobasal degeneration. Created with BioRender.com. Villa, C. (2026) https://BioRender.com/468n092, accessed on 30 March 2026.

Viewed together, the evidence discussed supports a multilevel model in which MBI domains may emerge as the clinical expression of selective network vulnerability in tauopathies. At the molecular level, tau misfolding, phosphorylation, and strain-specific propagation can disrupt microtubule stability, axonal transport, synaptic integrity, and neuromodulatory signaling, while glial and inflammatory responses may further amplify circuit instability. At the systems level, these processes might preferentially affect frontolimbic, salience, default mode, and frontostriatal networks that subserve motivation, emotional regulation, behavioral inhibition, and social cognition. The resulting network disconnection may then be phenotypically captured as the core MBI domains: decreased motivation arising predominantly from cingulofrontal and frontostriatal dysfunction, affective dysregulation and impulse dyscontrol from salience and orbitofrontal network disruption, and social inappropriateness from degeneration of socioemotional frontotemporal circuits. In this integrative perspective, MBI can be conceptualized as a domain-based clinical marker of early tau-related circuit failure across the spectrum of tauopathies (Figure 3).

## 6. Potential Therapeutic Candidates Targeting MBI

Given the increasing amount of evidence supporting the association between MBI and dementia, it can be hypothesized that treatment strategies targeting mild NPSs in subjects with normal cognition or MCI would be effective, particularly in reducing the risk of developing subsequent cognitive decline. However, future studies are necessary to determine whether MBI is a reversible condition [6]. Although no approved drug exists that specifically targets MBI, numerous pharmacological compounds as well as non-pharmacological approaches have shown potential benefits.

Among them, citalopram, a selective serotonin reuptake inhibitor (SSRI), has been reported to exhibit neuroprotective effects by promoting neurogenesis, suppressing neuroinflammation, and lowering amyloid and tau pathology [110]. SSRI treatment in patients with MCI and depression has also been associated with delayed progression to AD [111]. The use of cholinesterase inhibitors, specifically donepezil, has improved both NPSs and cognitive symptoms, particularly in patients with severe AD [112,113,114]. Noradrenergic and norepinephrine blocking agents, like prazosin and methylphenidate, were also found to be effective in alleviating apathy, agitation, and aggressiveness [115,116]. Moreover, other pharmacological compounds were explored showing promising results. In particular, phosphodiesterase-4 (PDE4) inhibitors, such as rolipram and roflumilast, have been shown to improve both cognitive function and depression in animal models, possibly by modulating the hypothalamic–pituitary–adrenal (HPA) axis, the cyclic AMP response-element-binding protein (CREB)/BDNF signaling pathway as well as anti-inflammatory mechanisms [117,118,119,120]. Additional agents, including melatonin, apelin-13, nattokinase, the antibiotic minocycline, and the ATP-sensitive potassium-channel inhibitor glibenclamide, reported a promising therapeutic potential, particularly in the domains of affective dysregulation and apathy in AD, through the regulation of HPA axis and neuroinflammatory responses [121,122,123,124,125]. Combined therapies, including antidepressant and acetylcholinesterase inhibitors, may further improve clinical outcomes, especially when mood disorders and cognitive impairment are diagnosed [126].

Notably, non-pharmacological interventions, like physical exercise and swimming, have also been linked to a lower risk of dementia by decreasing tau hyperphosphorylation, reducing amyloid deposition, preserving synaptic density, as well as suppressing neuroinflammation and neuronal damage [127]. Furthermore, these approaches have demonstrated promising results in ameliorating both cognitive deficiencies and behavioral symptoms, such as anxiety and depression [128,129,130].

## 7. Future Directions and Translational Implications

Despite the growing promise of MBI as an early behavioral framework across tauopathies, several conceptual and methodological challenges remain. While the MBI-C has brought standardization, the retrospective application of the MBI construct in previous datasets, such as using NPI data, has several limitations, including the shorter time frame, and the specificity of NPI mainly for dementia. As a result, symptom duration thresholds, domain boundaries, and cross-study comparability remain imperfect. In frontotemporal and other non-AD tauopathy cohorts, the optimal MBI-C cutoffs for case identification, prognostic enrichment, and differential diagnosis also remain insufficiently established.

Importantly, recent works highlight that scalable blood-based plasma biomarkers may support the study of MBI across the continuum of neurodegenerative diseases. In the early stages, diagnostic biomarkers may potentially help discriminate NPSs in the context of neurodegeneration from primary psychiatric syndromes and identifying the underlying neuropathology, whereas in dementia-stage disease, biomarkers might help track symptom trajectories and treatment responses [5,131]. In this context, growing evidence links MBI status and/or specific MBI domains with plasma biomarkers across the cognitive continuum, including associations with lower plasma Aβ42/40, longitudinal increases in plasma p-tau181, and higher plasma p-tau217 in dementia-free older adults [5,131]. These findings support the hypothesis that the combined use of MBI and plasma biomarkers might help in the differential diagnosis of the underlying neuropathology, especially in discriminating between AD and non-AD diseases. From a practical standpoint, MBI screening could serve as a low-cost triage step to select dementia-free individuals for confirmatory AD biomarker testing, such as p-tau217, potentially improving efficiency in prevention-oriented cohorts and trials [132,133].

The differentiation of MBI from primary psychiatric conditions remains difficult, particularly in younger patients, thereby resulting in potential diagnostic misclassification. Novel biomarkers, such as NfL may help towards this direction. In this regard, plasma NfL has shown high discriminatory performance in differentiating bvFTD from primary psychiatric disorders, such as bipolar disorder, major depression, or treatment-resistant schizophrenia [134]. In another study, serum NfL also differentiated bvFTD from primary psychiatric disorders with very good sensitivity and specificity, supporting the robustness of NfL across settings and cohorts [135]. This is highly relevant to the MBI framework, as many bvFTD presentations initially meet MBI criteria before overt dementia. Hence, combining MBI-based phenotyping with NfL could help flag neurodegeneration in late-onset psychiatric presentations that otherwise risk diagnostic delay. For astroglial markers, evidence suggests GFAP differs across etiologies and is not as strong as NfL for distinguishing bvFTD from primary psychiatric disorders. Plasma GFAP has been shown to be higher in bvFTD, but still inferior to NfL [134]. In broader FTD biomarker work, plasma GFAP has been found elevated in FTD versus cognitively normal controls, but lower than in AD [136]. Higher baseline GFAP has also been associated with faster longitudinal cognitive decline and increased risk of progression to cognitive impairment in FTD [136]. Finally, regarding tau-related markers, plasma total tau has been reported to be higher in clinical FTD syndromes, including bvFTD, versus healthy controls, but shows genetic subtype effects, with significant increases in symptomatic *MAPT* mutation carriers, and not consistently in *C9orf72* or *GRN* [137]. Taken together, these findings support a biomarker-informed approach in which MBI-based behavioral phenotyping, particularly when combined with plasma NfL and selected glial or tau-related markers, may improve the early distinction between prodromal neurodegeneration and primary psychiatric disease.

Furthermore, despite behavioral symptoms being common, many neurodegenerative disease trials still exclude participants with significant psychiatric symptoms, paradoxically omitting those most relevant to the MBI construct. While predictive associations for MBI symptoms are relatively strong in AD-related cohorts, large-scale, prospective studies in other tauopathies are limited. These limitations reinforce the need for refined instruments and cutoff points, more uniform stratification methods, and more inclusive research designs.

Several priorities should shape the next phase of translational research. First, prospective longitudinal studies are needed to validate MBI domains against multimodal biomarkers, including plasma and CSF NfL, tau species and isoform-sensitive assays, inflammatory markers, and structural and functional network imaging. Such studies should move beyond total MBI burden and examine whether specific domain profiles map onto distinct molecular signatures, patterns of network disintegration, and rates of progression. Second, genotype-informed phenotyping may help define mutation-specific prodromal behavioral trajectories and clarify whether MBI can serve as a trans-syndromic but biologically informative marker of underlying pathology. Third, domain-level neuroimaging approaches should examine whether decreased motivation, affective dysregulation, impulse dyscontrol, and social inappropriateness can be reliably linked to selective disruption of frontostriatal, salience, default mode, or socioemotional networks before overt dementia emerges. Fourth, digital phenotyping offers a major opportunity for scale: passive behavioral monitoring, speech and language analysis, actigraphy, social interaction metrics, facial affect recognition, and ecologically sampled behavioral data may help operationalize MBI continuously and sensitively outside the clinic. Finally, MBI should be incorporated into experimental medicine and early-phase intervention trials, not only as a screening or enrichment tool, but also as a potential outcome measure for therapies targeting synaptic dysfunction, network instability, neuroinflammation, or pathogenic protein spread. In this context, MBI may ultimately contribute to a more biologically grounded stratification of neurodegenerative disease, refine subtype classification within existing diagnostic categories, and support earlier, more personalized behavioral and disease-modifying interventions.

## 8. Conclusions—Reframing Behavior in Neurodegeneration

MBI offers a clinically meaningful framework for reinterpreting later-life-emergent NPSs as potential early manifestations of neurodegenerative disease. Across the spectrum of tauopathies, converging clinical, neuroimaging, molecular, and biomarker evidence suggests that persistent behavioral change may reflect the selective vulnerability of frontolimbic, salience, default mode, and frontostriatal networks to tau-mediated dysfunction. In this context, MBI domains can be viewed as a useful marker of early circuit failure, linking symptoms such as apathy, affective dysregulation, impulsivity, disinhibition, and altered social conduct to underlying synaptic, neuromodulatory, inflammatory, and network-level pathology. This perspective is particularly valuable in disorders such as AD, bvFTD, PSP, and CBD, where behavioral symptoms may precede overt dementia or major motor syndromes and therefore provide an important window into prodromal disease.

At a translational level, MBI has the potential to serve as a link between bedside phenotyping and biomarker-based neurodegeneration research. When integrated with fluid biomarkers, genotype-informed stratification, and multimodal neuroimaging, MBI may improve early detection, refine differential diagnosis, enrich clinical trial populations, and offer domain-sensitive outcome measures for prevention-oriented and disease-modifying interventions. Reframing behavior in this way shifts NPSs from the periphery to the center of neurodegenerative disease models. Recognizing and operationalizing MBI as the possibly earliest clinically accessible expression of tau-related brain dysfunction may help towards advancing earlier, biologically informed, and more personalized approaches to the spectrum of tauopathies.

## Figures and Tables

**Table 1:** A summary of main genetic factors associated with mild behavioral impairment.

Genetic Factors	MBI Domains	References
*APOE* ε4	↑ Affective dysregulation	[18]
*MS4A4A*/*MS4A6A*	↓ Affective dysregulation	[18]
*ZCWPW1*	↓ Social inappropriateness and psychosis	[18]
*EPHA1*	↑ Psychosis	[18]
*BIN1*	↑ Psychosis	[18]
*NME8*	↓ Apathy	[18]
*BDNF*	↑ Affective dysregulation and psychosis	[19]

## Data Availability

No new data were created or analyzed in this study.

## Abbreviations

The following abbreviations are used in this manuscript:

AD	Alzheimer’s disease
ALS	amyotrophic lateral sclerosis
aMCI	amnestic MCI
AVLT	auditory verbal learning test
BNT	Boston naming test
BPSD	behavioral and psychological symptoms of dementia
bvFTD	behavioral variant FTD
CBD	corticobasal degeneration
CBS	corticobasal syndrome
CDR	Clinical Dementia Rating
CREB	cyclic AMP response-element-binding protein
CSF	cerebrospinal fluid
DAT	dopamine transporter
DRN	dorsal raphe nucleus
FBI	Frontal Behavioral Inventory
FTD	frontotemporal dementia
FTLD	frontotemporal lobar degeneration
FTLD-tau	frontotemporal lobar degeneration with tau pathology
FTSD	frontotemporal spectrum disorders
GDS-15	geriatric depression scale-15
GENFI	genetic FTD initiative
HPA	hypothalamic–pituitary–adrenal
HT	hydroxytryptamine
IL	interleukin
ISTAART-AA	international society to advance Alzheimer’s research and treatment-Alzheimer’s association
LC	locus coeruleus
MBI	mild behavioral impairment
MBI-C	MBI checklist
MCI	mild cognitive impairment
MNDs	motor neuron diseases
NF-κB	nuclear factor kappa-light-chain-enhancer of activated B cells
NfL	neurofilament light
NFTs	neurofibrillary tangles
NPI	Neuropsychiatric Inventory
NPI-Q	NPI-questionnaire
NPS	neuropsychiatric symptoms
PD	Parkinson’s disease
PDE4	phosphodiesterase-4
PET	positron emission tomography
PIA	professional interest area
PSP	supranuclear palsy
SCD	subjective cognitive decline
SERT	serotonin reuptake transporter
SSRI	selective serotonin reuptake inhibitor
SV2A	synaptic vesicle glycoprotein 2A
TMT-A	trail making test part A
TMT-B	trail making test part B
TREM2	triggering receptor expressed on myeloid cells 2
VAChT	vesicular acetylcholine transporter

## References

[1] Ismail Z., Agüera-Ortiz L., Brodaty H., Cieslak A., Cummings J., Fischer C.E., Gauthier S., Geda Y.E., Herrmann N., Kanji J. (2017). The Mild Behavioral Impairment Checklist (MBI-C): A Rating Scale for Neuropsychiatric Symptoms in Pre-Dementia Populations. J. Alzheimers Dis..

[2] Ismail Z., Smith E.E., Geda Y., Sultzer D., Brodaty H., Smith G., Agüera-Ortiz L., Sweet R., Miller D., Lyketsos C.G. (2016). Neuropsychiatric symptoms as early manifestations of emergent dementia: Provisional diagnostic criteria for mild behavioral impairment. Alzheimers Dement..

[3] Cummings J. (2021). The Role of Neuropsychiatric Symptoms in Research Diagnostic Criteria for Neurodegenerative Diseases. Am. J. Geriatr. Psychiatry.

[4] Creekmore B.C., Watanabe R., Lee E.B. (2024). Neurodegenerative Disease Tauopathies. Annu. Rev. Pathol..

[5] Angelopoulou E., Androni X., Villa C., Hatzimanolis A., Scarmeas N., Papageorgiou S. (2025). Blood-based biomarkers in mild behavioral impairment: An updated overview. Front. Neurol..

[6] Angelopoulou E., Bougea A., Hatzimanolis A., Scarmeas N., Papageorgiou S.G. (2024). Unraveling the Potential Underlying Mechanisms of Mild Behavioral Impairment: Focusing on Amyloid and Tau Pathology. Cells.

[7] Angelopoulou E., Koros C., Hatzimanolis A., Stefanis L., Scarmeas N., Papageorgiou S.G. (2024). Exploring the Genetic Landscape of Mild Behavioral Impairment as an Early Marker of Cognitive Decline: An Updated Review Focusing on Alzheimer’s Disease. Int. J. Mol. Sci..

[8] Johansson M., Stomrud E., Insel P.S., Leuzy A., Johansson P.M., Smith R., Ismail Z., Janelidze S., Palmqvist S., van Westen D. (2021). Mild behavioral impairment and its relation to tau pathology in preclinical Alzheimer’s disease. Transl. Psychiatry.

[9] Iordan A.D., Ploutz-Snyder R., Ghosh B., Rahman-Filipiak A., Koeppe R., Peltier S., Giordani B., Albin R.L., Hampstead B.M. (2024). Salience network segregation mediates the effect of tau pathology on mild behavioral impairment. Alzheimers Dement..

[10] Pinilla-Monsalve G.D., Song Y.P., Hanganu A., Ismail Z., Monchi O. (2026). The Mild Behavioral Impairment Checklist for Parkinson’s Disease: An Ancillary Instrument in Cognitive Assessment. Mov. Disord. Clin. Pract..

[11] Duranti E., Villa C. (2026). Misfolded Proteins and Cognitive Decline: Mechanistic Insights into Neurodegenerative Disorders. Neurol. Int..

[12] Spisto M., Moretta P., Senerchia G., Iuzzolino V.V., Aruta L., Salvatore E., Santangelo G., Trojano L., Dubbioso R. (2025). Identifying Mild Behavioral and Neurocognitive Impairment in Amyotrophic Lateral Sclerosis (MBNI-ALS) Provides Key Prognostic Insights. Eur. J. Neurol..

[13] Ferraro P.M., Gervino E., De Maria E., Meo G., Ponzano M., Pardini M., Signori A., Schenone A., Roccatagliata L., Caponnetto C. (2023). Mild behavioral impairment as a potential marker of predementia risk states in motor neuron diseases. Eur. J. Neurol..

[14] Hu S., Patten S., Charlton A., Fischer K., Fick G., Smith E.E., Ismail Z. (2023). Validating the Mild Behavioral Impairment Checklist in a Cognitive Clinic: Comparisons with the Neuropsychiatric Inventory Questionnaire. J. Geriatr. Psychiatry Neurol..

[15] Mallo S.C., Ismail Z., Pereiro A.X., Facal D., Lojo-Seoane C., Campos-Magdaleno M., Juncos-Rabadán O. (2019). Assessing mild behavioral impairment with the mild behavioral impairment checklist in people with subjective cognitive decline. Int. Psychogeriatr..

[16] Kan C.N., Cano J., Zhao X., Ismail Z., Chen C.L., Xu X. (2022). Prevalence, Clinical Correlates, Cognitive Trajectories, and Dementia Risk Associated with Mild Behavioral Impairment in Asians. J. Clin. Psychiatry.

[17] Sun Y., Xu W., Chen K.L., Shen X.N., Tan L., Yu J.T. (2021). Mild behavioral impairment correlates of cognitive impairments in older adults without dementia: Mediation by amyloid pathology. Transl. Psychiatry.

[18] Andrews S.J., Ismail Z., Anstey K.J., Mortby M. (2018). Association of Alzheimer’s genetic loci with mild behavioral impairment. Am. J. Med. Genet. B Neuropsychiatr. Genet..

[19] Ramezani M., Ruskey J.A., Martens K., Kibreab M., Javer Z., Kathol I., Hammer T., Cheetham J., Leveille E., Martino D. (2020). Association Between BDNF Val66Met Polymorphism and Mild Behavioral Impairment in Patients With Parkinson’s Disease. Front. Neurol..

[20] Guan D.X., Smith E.E., Pike G.B., Ismail Z. (2023). Persistence of neuropsychiatric symptoms and dementia prognostication: A comparison of three operational case definitions of mild behavioral impairment. Alzheimers Dement..

[21] Angelopoulou E., Stanitsa E., Hatzopoulou M., Despoti A., Tsinia N., Kamtsadeli V., Papadogiani M., Kyriakidis V., Papageorgiou S., Papatriantafyllou J.D. (2025). The Greek Version of the Mild Behavioral Impairment Checklist (MBI-C): Psychometric Properties in Mild Cognitive Impairment Due to Alzheimer’s Disease. Brain Sci..

[22] Iqbal K., Liu F., Gong C.X. (2016). Tau and neurodegenerative disease: The story so far. Nat. Rev. Neurol..

[23] Matuskova V., Veverova K., Jester D.J., Matoska V., Ismail Z., Sheardova K., Horakova H., Cerman J., Laczó J., Andel R. (2024). Mild behavioral impairment in early Alzheimer’s disease and its association with APOE and BDNF risk genetic polymorphisms. Alzheimers Res. Ther..

[24] Naude J., Wang M., Leon R., Smith E., Ismail Z. (2024). Tau-PET in early cortical Alzheimer brain regions in relation to mild behavioral impairment in older adults with either normal cognition or mild cognitive impairment. Neurobiol. Aging.

[25] Ghahremani M., Wang M., Chen H.Y., Zetterberg H., Smith E., Ismail Z. (2023). Plasma Phosphorylated Tau at Threonine 181 and Neuropsychiatric Symptoms in Preclinical and Prodromal Alzheimer Disease. Neurology.

[26] Gonzalez-Bautista E., Momméja M., de Mauléon A., Ismail Z., Vellas B., Delrieu J., Soto Martin M.E. (2024). Mild behavioral impairment domains are longitudinally associated with pTAU and metabolic biomarkers in dementia-free older adults. Alzheimers Dement..

[27] Ismail Z., Leon R., Creese B., Ballard C., Robert P., Smith E.E. (2023). Optimizing detection of Alzheimer’s disease in mild cognitive impairment: A 4-year biomarker study of mild behavioral impairment in ADNI and MEMENTO. Mol. Neurodegener..

[28] Leon R., Ghahremani M., Guan D.X., Smith E.E., Zetterberg H., Ismail Z. (2026). Enhancing Alzheimer Disease Detection Using Neuropsychiatric Symptoms: The Role of Mild Behavioural Impairment in the Revised NIA-AA Research Framework. J. Geriatr. Psychiatry Neurol..

[29] Badhwar A., Tam A., Dansereau C., Orban P., Hoffstaedter F., Bellec P. (2017). Resting-state network dysfunction in Alzheimer’s disease: A systematic review and meta-analysis. Alzheimers Dement..

[30] Vellone D., Leon R., Goodarzi Z., Forkert N.D., Smith E.E., Ismail Z. (2025). Mild behavioural impairment-apathy and core Alzheimer’s disease cerebrospinal fluid biomarkers. Brain.

[31] Rascovsky K., Hodges J.R., Knopman D., Mendez M.F., Kramer J.H., Neuhaus J., van Swieten J.C., Seelaar H., Dopper E.G., Onyike C.U. (2011). Sensitivity of revised diagnostic criteria for the behavioural variant of frontotemporal dementia. Brain.

[32] Taragano F.E., Allegri R.F., Krupitzki H., Sarasola D.R., Serrano C.M., Loñ L., Lyketsos C.G. (2009). Mild behavioral impairment and risk of dementia: A prospective cohort study of 358 patients. J. Clin. Psychiatry.

[33] Schölzel-Dorenbos C.J. (2006). Mild behavioral impairment: A prodromal stage of frontotemporal lobar degeneration. J. Am. Geriatr. Soc..

[34] Taragano F.E., Allegri R.F., Lyketsos C. (2008). Mild behavioral impairment: A prodromal stage of dementia. Dement. Neuropsychol..

[35] Orso B., Mattei C., Arnaldi D., Massa F., Serafini G., Plantone D., Doglione E., Grafman J., Nobili F., Pardini M. (2020). Clinical and MRI Predictors of Conversion from Mild Behavioural Impairment to Dementia. Am. J. Geriatr. Psychiatry.

[36] Taragano F.E., Allegri R.F., Heisecke S.L., Martelli M.I., Feldman M.L., Sánchez V., García V.A., Tufro G., Castro D.M., Leguizamón P.P. (2018). Risk of Conversion to Dementia in a Mild Behavioral Impairment Group Compared to a Psychiatric Group and to a Mild Cognitive Impairment Group. J. Alzheimers Dis..

[37] Kertesz A., Davidson W., Fox H. (1997). Frontal behavioral inventory: Diagnostic criteria for frontal lobe dementia. Can. J. Neurol. Sci..

[38] Cheran G., Silverman H., Manoochehri M., Goldman J., Lee S., Wu L., Cines S., Fallon E., Kelly B.D., Olszewska D.A. (2018). Psychiatric symptoms in preclinical behavioural-variant frontotemporal dementia in MAPT mutation carriers. J. Neurol. Neurosurg. Psychiatry.

[39] Cui Y., Liu L., Chu M., Xie K., Chen Z., Nan H., Kong Y., Xia T., Wang Y., He Q. (2024). Application of the mild behavioral impairment checklist in Chinese patients with the behavioral variant of frontotemporal dementia. Neurol. Sci..

[40] Cieslak A., Smith E.E., Lysack J., Ismail Z. (2018). Case series of mild behavioral impairment: Toward an understanding of the early stages of neurodegenerative diseases affecting behavior and cognition. Int. Psychogeriatr..

[41] Cui Y., Dai S., Miao Z., Zhong Y., Liu Y., Liu L., Jing D., Bai Y., Kong Y., Sun W. (2019). Reliability and Validity of the Chinese Version of the Mild Behavioral Impairment Checklist for Screening for Alzheimer’s Disease. J. Alzheimers Dis..

[42] Dodich A., Cerami C., Crespi C., Canessa N., Lettieri G., Iannaccone S., Marcone A., Cappa S.F., Cacioppo J.T. (2016). Differential Impairment of Cognitive and Affective Mentalizing Abilities in Neurodegenerative Dementias: Evidence from Behavioral Variant of Frontotemporal Dementia, Alzheimer’s Disease, and Mild Cognitive Impairment. J. Alzheimers Dis..

[43] Gonçalves S.A.B., Caramelli P., Mariano L.I., Guimarães H.C., Gambogi L.B., Resende E.P.F., Teixeira A.L., de Souza L.C. (2020). Apathy in frontotemporal dementia is related to medial prefrontal atrophy and is independent of executive dysfunction. Brain Res..

[44] Sheelakumari R., Bineesh C., Varghese T., Kesavadas C., Verghese J., Mathuranath P.S. (2020). Neuroanatomical correlates of apathy and disinhibition in behavioural variant frontotemporal dementia. Brain Imaging Behav..

[45] Whitwell J.L., Przybelski S.A., Weigand S.D., Ivnik R.J., Vemuri P., Gunter J.L., Senjem M.L., Shiung M.M., Boeve B.F., Knopman D.S. (2009). Distinct anatomical subtypes of the behavioural variant of frontotemporal dementia: A cluster analysis study. Brain.

[46] Rankin K.P., Kramer J.H., Miller B.L. (2005). Patterns of cognitive and emotional empathy in frontotemporal lobar degeneration. Cogn. Behav. Neurol..

[47] Ducharme S., Dols A., Laforce R., Devenney E., Kumfor F., van den Stock J., Dallaire-Théroux C., Seelaar H., Gossink F., Vijverberg E. (2020). Recommendations to distinguish behavioural variant frontotemporal dementia from psychiatric disorders. Brain.

[48] Tavares T.P., Mitchell D.G.V., Coleman K.K., Coleman B.L., Shoesmith C.L., Butler C.R., Santana I., Danek A., Gerhard A., de Mendonca A. (2020). Early symptoms in symptomatic and preclinical genetic frontotemporal lobar degeneration. J. Neurol. Neurosurg. Psychiatry.

[49] Benussi A., Premi E., Gazzina S., Brattini C., Bonomi E., Alberici A., Jiskoot L., van Swieten J.C., Sanchez-Valle R., Moreno F. (2021). Progression of Behavioral Disturbances and Neuropsychiatric Symptoms in Patients with Genetic Frontotemporal Dementia. JAMA Netw. Open.

[50] Samra K., Macdougall A., Peakman G., Bouzigues A., Bocchetta M., Cash D.M., Greaves C.V., Convery R.S., van Swieten J.C., Jiskoot L.C. (2023). Neuropsychiatric symptoms in genetic frontotemporal dementia: Developing a new module for Clinical Rating Scales. J. Neurol. Neurosurg. Psychiatry.

[51] Malpetti M., Jones P.S., Tsvetanov K.A., Rittman T., van Swieten J.C., Borroni B., Sanchez-Valle R., Moreno F., Laforce R., Graff C. (2021). Apathy in presymptomatic genetic frontotemporal dementia predicts cognitive decline and is driven by structural brain changes. Alzheimers Dement..

[52] Jackson R.J., Melloni A., Fykstra D.P., Serrano-Pozo A., Shinobu L., Hyman B.T. (2024). Astrocyte tau deposition in progressive supranuclear palsy is associated with dysregulation of MAPT transcription. Acta Neuropathol. Commun..

[53] Illán-Gala I., Nigro S., VandeVrede L., Falgàs N., Heuer H.W., Painous C., Compta Y., Martí M.J., Montal V., Pagonabarraga J. (2022). Diagnostic Accuracy of Magnetic Resonance Imaging Measures of Brain Atrophy Across the Spectrum of Progressive Supranuclear Palsy and Corticobasal Degeneration. JAMA Netw. Open.

[54] Flavell J., Nestor P.J. (2022). A Systematic Review of Apathy and Depression in Progressive Supranuclear Palsy. J. Geriatr. Psychiatry Neurol..

[55] Borroni B., Alberici A., Agosti C., Cosseddu M., Padovani A. (2009). Pattern of behavioral disturbances in corticobasal degeneration syndrome and progressive supranuclear palsy. Int. Psychogeriatr..

[56] Kok Z.Q., Murley A.G., Rittman T., Rowe J., Passamonti L. (2021). Co-Occurrence of Apathy and Impulsivity in Progressive Supranuclear Palsy. Mov. Disord. Clin. Pract..

[57] Josephs K.A., Whitwell J.L., Eggers S.D., Senjem M.L., Jack C.R. (2011). Gray matter correlates of behavioral severity in progressive supranuclear palsy. Mov. Disord..

[58] Ye R., O’Callaghan C., Rua C., Hezemans F.H., Holland N., Malpetti M., Jones P.S., Barker R.A., Williams-Gray C.H., Robbins T.W. (2022). Locus Coeruleus Integrity from 7 T MRI Relates to Apathy and Cognition in Parkinsonian Disorders. Mov. Disord..

[59] Geda Y.E., Boeve B.F., Negash S., Graff-Radford N.R., Knopman D.S., Parisi J.E., Dickson D.W., Petersen R.C. (2007). Neuropsychiatric features in 36 pathologically confirmed cases of corticobasal degeneration. J. Neuropsychiatry Clin. Neurosci..

[60] Shelley B.P., Hodges J.R., Kipps C.M., Xuereb J.H., Bak T.H. (2009). Is the pathology of corticobasal syndrome predictable in life?. Mov. Disord..

[61] Braak H., Braak E. (1991). Neuropathological stageing of Alzheimer-related changes. Acta Neuropathol..

[62] Hoenig M.C., Bischof G.N., Seemiller J., Hammes J., Kukolja J., Onur Ö.A., Jessen F., Fliessbach K., Neumaier B., Fink G.R. (2018). Networks of tau distribution in Alzheimer’s disease. Brain.

[63] Guo J.L., Lee V.M. (2011). Seeding of normal Tau by pathological Tau conformers drives pathogenesis of Alzheimer-like tangles. J. Biol. Chem..

[64] Clavaguera F., Bolmont T., Crowther R.A., Abramowski D., Frank S., Probst A., Fraser G., Stalder A.K., Beibel M., Staufenbiel M. (2009). Transmission and spreading of tauopathy in transgenic mouse brain. Nat. Cell Biol..

[65] Katsumi Y., Howe I.A., Eckbo R., Wong B., Quimby M., Hochberg D., McGinnis S.M., Putcha D., Wolk D.A., Touroutoglou A. (2025). Default mode network tau predicts future clinical decline in atypical early Alzheimer’s disease. Brain.

[66] Seeley W.W., Crawford R.K., Zhou J., Miller B.L., Greicius M.D. (2009). Neurodegenerative diseases target large-scale human brain networks. Neuron.

[67] Schimmelpfennig J., Topczewski J., Zajkowski W., Jankowiak-Siuda K. (2023). The role of the salience network in cognitive and affective deficits. Front. Hum. Neurosci..

[68] Azarias F.R., Almeida G., de Melo L.F., Rici R.E.G., Maria D.A. (2025). The Journey of the Default Mode Network: Development, Function, and Impact on Mental Health. Biology.

[69] Nishioka C., Liang H.F., Ong S., Sun S.W. (2022). Axonal Transport Impairment and its Relationship with Diffusion Tensor Imaging Metrics of a Murine Model of p301L Tau Induced Tauopathy. Neuroscience.

[70] Colom-Cadena M., Davies C., Sirisi S., Lee J.E., Simzer E.M., Tzioras M., Querol-Vilaseca M., Sánchez-Aced É., Chang Y.Y., Holt K. (2023). Synaptic oligomeric tau in Alzheimer’s disease—A potential culprit in the spread of tau pathology through the brain. Neuron.

[71] Braak H., Thal D.R., Ghebremedhin E., Del Tredici K. (2011). Stages of the pathologic process in Alzheimer disease: Age categories from 1 to 100 years. J. Neuropathol. Exp. Neurol..

[72] Sara S.J. (2009). The locus coeruleus and noradrenergic modulation of cognition. Nat. Rev. Neurosci..

[73] Braak H., Del Tredici K. (2015). The preclinical phase of the pathological process underlying sporadic Alzheimer’s disease. Brain.

[74] Ehrenberg A.J., Nguy A.K., Theofilas P., Dunlop S., Suemoto C.K., Di Lorenzo Alho A.T., Leite R.P., Diehl Rodriguez R., Mejia M.B., Rüb U. (2017). Quantifying the accretion of hyperphosphorylated tau in the locus coeruleus and dorsal raphe nucleus: The pathological building blocks of early Alzheimer’s disease. Neuropathol. Appl. Neurobiol..

[75] Grinberg L.T., Rüb U., Ferretti R.E., Nitrini R., Farfel J.M., Polichiso L., Gierga K., Jacob-Filho W., Heinsen H. (2009). The dorsal raphe nucleus shows phospho-tau neurofibrillary changes before the transentorhinal region in Alzheimer’s disease. A precocious onset?. Neuropathol. Appl. Neurobiol..

[76] Pierson S.R., Fiock K.L., Wang R., Balasubramanian N., Reinhardt J., Khan K.M., James T.D., Hunter M.L., Cooper B.J., Williamsen H.R. (2025). Tau pathology in the dorsal raphe may be a prodromal indicator of Alzheimer’s disease. Mol. Psychiatry.

[77] Hu W., Zhang X., Tung Y.C., Xie S., Liu F., Iqbal K. (2016). Hyperphosphorylation determines both the spread and the morphology of tau pathology. Alzheimers Dement..

[78] Goedert M., Falcon B., Clavaguera F., Tolnay M. (2014). Prion-like mechanisms in the pathogenesis of tauopathies and synucleinopathies. Curr. Neurol. Neurosci. Rep..

[79] Ayers J.I., Giasson B.I., Borchelt D.R. (2018). Prion-like Spreading in Tauopathies. Biol. Psychiatry.

[80] Irwin D.J., Brettschneider J., McMillan C.T., Cooper F., Olm C., Arnold S.E., Van Deerlin V.M., Seeley W.W., Miller B.L., Lee E.B. (2016). Deep clinical and neuropathological phenotyping of Pick disease. Ann. Neurol..

[81] Gossye H., Van Mossevelde S., Sieben A., Bjerke M., Hendrickx Van de Craen E., van der Zee J., De Deyn P.P., De Bleecker J., Versijpt J., van den Ende J. (2023). Patients carrying the mutation p.R406W in MAPT present with non-conforming phenotypic spectrum. Brain.

[82] Ghosh B.C., Calder A.J., Peers P.V., Lawrence A.D., Acosta-Cabronero J., Pereira J.M., Hodges J.R., Rowe J.B. (2012). Social cognitive deficits and their neural correlates in progressive supranuclear palsy. Brain.

[83] Lee S.E., Rabinovici G.D., Mayo M.C., Wilson S.M., Seeley W.W., DeArmond S.J., Huang E.J., Trojanowski J.Q., Growdon M.E., Jang J.Y. (2011). Clinicopathological correlations in corticobasal degeneration. Ann. Neurol..

[84] Sakae N., Santos O.A., Pedraza O., Litvan I., Murray M.E., Duara R., Uitti R.J., Wszolek Z.K., Graff-Radford N.R., Josephs K.A. (2020). Clinical and pathologic features of cognitive-predominant corticobasal degeneration. Neurology.

[85] Mecca A.P., Chen M.K., O’Dell R.S., Naganawa M., Toyonaga T., Godek T.A., Harris J.E., Bartlett H.H., Zhao W., Nabulsi N.B. (2020). In vivo measurement of widespread synaptic loss in Alzheimer’s disease with SV2A PET. Alzheimers Dement..

[86] Malpetti M., Jones P.S., Cope T.E., Holland N., Naessens M., Rouse M.A., Rittman T., Savulich G., Whiteside D.J., Street D. (2023). Synaptic Loss in Frontotemporal Dementia Revealed by [(11) C]UCB-J Positron Emission Tomography. Ann. Neurol..

[87] Holland N., Jones P.S., Savulich G., Wiggins J.K., Hong Y.T., Fryer T.D., Manavaki R., Sephton S.M., Boros I., Malpetti M. (2020). Synaptic Loss in Primary Tauopathies Revealed by [(11) C]UCB-J Positron Emission Tomography. Mov. Disord..

[88] Murley A.G., Rowe J.B. (2018). Neurotransmitter deficits from frontotemporal lobar degeneration. Brain.

[89] Lewis R.G., Florio E., Punzo D., Borrelli E. (2021). The Brain’s Reward System in Health and Disease. Circadian clock in brain health and disease.

[90] Kobayashi R., Kawakatsu S., Ohba M., Morioka D., Kanoto M., Otani K. (2022). Dopamine Transporter Imaging for Frontotemporal Lobar Degeneration With Motor Neuron Disease. Front. Neurosci..

[91] Huber N., Korhonen S., Hoffmann D., Leskelä S., Rostalski H., Remes A.M., Honkakoski P., Solje E., Haapasalo A. (2022). Deficient neurotransmitter systems and synaptic function in frontotemporal lobar degeneration-Insights into disease mechanisms and current therapeutic approaches. Mol. Psychiatry.

[92] Asai H., Ikezu S., Tsunoda S., Medalla M., Luebke J., Haydar T., Wolozin B., Butovsky O., Kügler S., Ikezu T. (2015). Depletion of microglia and inhibition of exosome synthesis halt tau propagation. Nat. Neurosci..

[93] Wang C., Fan L., Khawaja R.R., Liu B., Zhan L., Kodama L., Chin M., Li Y., Le D., Zhou Y. (2022). Microglial NF-κB drives tau spreading and toxicity in a mouse model of tauopathy. Nat. Commun..

[94] Leyns C.E.G., Ulrich J.D., Finn M.B., Stewart F.R., Koscal L.J., Remolina Serrano J., Robinson G.O., Anderson E., Colonna M., Holtzman D.M. (2017). TREM2 deficiency attenuates neuroinflammation and protects against neurodegeneration in a mouse model of tauopathy. Proc. Natl. Acad. Sci. USA.

[95] Ising C., Venegas C., Zhang S., Scheiblich H., Schmidt S.V., Vieira-Saecker A., Schwartz S., Albasset S., McManus R.M., Tejera D. (2019). NLRP3 inflammasome activation drives tau pathology. Nature.

[96] Litvinchuk A., Wan Y.W., Swartzlander D.B., Chen F., Cole A., Propson N.E., Wang Q., Zhang B., Liu Z., Zheng H. (2018). Complement C3aR Inactivation Attenuates Tau Pathology and Reverses an Immune Network Deregulated in Tauopathy Models and Alzheimer’s Disease. Neuron.

[97] Rohden F., Ferreira P.C.L., Bellaver B., Ferrari-Souza J.P., Aguzzoli C.S., Soares C., Abbas S., Zalzale H., Povala G., Lussier F.Z. (2025). Glial reactivity correlates with synaptic dysfunction across aging and Alzheimer’s disease. Nat. Commun..

[98] Taddei R.N., Perbet R., Mate de Gerando A., Wiedmer A.E., Sanchez-Mico M., Connors Stewart T., Gaona A., Melloni A., Amaral A.C., Duff K. (2023). Tau Oligomer-Containing Synapse Elimination by Microglia and Astrocytes in Alzheimer Disease. JAMA Neurol..

[99] Yoshida M. (2014). Astrocytic inclusions in progressive supranuclear palsy and corticobasal degeneration. Neuropathology.

[100] Kovacs G.G., Lukic M.J., Irwin D.J., Arzberger T., Respondek G., Lee E.B., Coughlin D., Giese A., Grossman M., Kurz C. (2020). Distribution patterns of tau pathology in progressive supranuclear palsy. Acta Neuropathol..

[101] Robinson J.L., Yan N., Caswell C., Xie S.X., Suh E., Van Deerlin V.M., Gibbons G., Irwin D.J., Grossman M., Lee E.B. (2020). Primary Tau Pathology, Not Copathology, Correlates with Clinical Symptoms in PSP and CBD. J. Neuropathol. Exp. Neurol..

[102] Huang S.Y., Chen S.F., Cui M., Zhao M., Shen X.N., Guo Y., Zhang Y.R., Zhang W., Wang H.F., Huang Y.Y. (2023). Plasma Biomarkers and Positron Emission Tomography Tau Pathology in Progressive Supranuclear Palsy. Mov. Disord..

[103] Fiock K.L., Hook J.N., Hefti M.M. (2023). Determinants of astrocytic pathology in stem cell models of primary tauopathies. Acta Neuropathol. Commun..

[104] McGeachan R.I., Keavey L., Simzer E.M., Chang Y.Y., Rose J.L., Spires-Jones M.P., Gilmore M., Holt K., Meftah S., Ravingerova N. (2025). Evidence for trans-synaptic propagation of oligomeric tau in human progressive supranuclear palsy. Nat. Neurosci..

[105] Hartnell I.J., Woodhouse D., Jasper W., Mason L., Marwaha P., Graffeuil M., Lau L.C., Norman J.L., Chatelet D.S., Buee L. (2024). Glial reactivity and T cell infiltration in frontotemporal lobar degeneration with tau pathology. Brain.

[106] Kim M.J., McGwier M., Jenko K.J., Snow J., Morse C., Zoghbi S.S., Pike V.W., Innis R.B., Kreisl W.C. (2019). Neuroinflammation in frontotemporal lobar degeneration revealed by (11) C-PBR28 PET. Ann. Clin. Transl. Neurol..

[107] Guerreiro R.J., Lohmann E., Brás J.M., Gibbs J.R., Rohrer J.D., Gurunlian N., Dursun B., Bilgic B., Hanagasi H., Gurvit H. (2013). Using exome sequencing to reveal mutations in TREM2 presenting as a frontotemporal dementia-like syndrome without bone involvement. JAMA Neurol..

[108] Johnson A.M., Lukens J.R. (2023). The innate immune response in tauopathies. Eur. J. Immunol..

[109] Wang C., Xiong M., Gratuze M., Bao X., Shi Y., Andhey P.S., Manis M., Schroeder C., Yin Z., Madore C. (2021). Selective removal of astrocytic APOE4 strongly protects against tau-mediated neurodegeneration and decreases synaptic phagocytosis by microglia. Neuron.

[110] Sawant N., Kshirsagar S., Reddy P.H., Reddy A.P. (2024). Protective effects of SSRI, Citalopram in mutant APP and mutant Tau expressed dorsal raphe neurons in Alzheimer’s disease. Biochim. Biophys. Acta Mol. Basis Dis..

[111] Bartels C., Wagner M., Wolfsgruber S., Ehrenreich H., Schneider A. (2018). Impact of SSRI Therapy on Risk of Conversion From Mild Cognitive Impairment to Alzheimer’s Dementia in Individuals With Previous Depression. Am. J. Psychiatry.

[112] Holmes C., Wilkinson D., Dean C., Vethanayagam S., Olivieri S., Langley A., Pandita-Gunawardena N.D., Hogg F., Clare C., Damms J. (2004). The efficacy of donepezil in the treatment of neuropsychiatric symptoms in Alzheimer disease. Neurology.

[113] Cummings J.L., McRae T., Zhang R. (2006). Effects of donepezil on neuropsychiatric symptoms in patients with dementia and severe behavioral disorders. Am. J. Geriatr. Psychiatry.

[114] Feldman H., Gauthier S., Hecker J., Vellas B., Xu Y., Ieni J.R., Schwam E.M. (2005). Efficacy and safety of donepezil in patients with more severe Alzheimer’s disease: A subgroup analysis from a randomized, placebo-controlled trial. Int. J. Geriatr. Psychiatry.

[115] Wang L.Y., Shofer J.B., Rohde K., Hart K.L., Hoff D.J., McFall Y.H., Raskind M.A., Peskind E.R. (2009). Prazosin for the treatment of behavioral symptoms in patients with Alzheimer disease with agitation and aggression. Am. J. Geriatr. Psychiatry.

[116] David M.C.B., Del Giovane M., Liu K.Y., Gostick B., Rowe J.B., Oboh I., Howard R., Malhotra P.A. (2022). Cognitive and neuropsychiatric effects of noradrenergic treatment in Alzheimer’s disease: Systematic review and meta-analysis. J. Neurol. Neurosurg. Psychiatry.

[117] Cong Y.F., Liu F.W., Xu L., Song S.S., Shen X.R., Liu D., Hou X.Q., Zhang H.T. (2023). Rolipram Ameliorates Memory Deficits and Depression-Like Behavior in APP/PS1/tau Triple Transgenic Mice: Involvement of Neuroinflammation and Apoptosis via cAMP Signaling. Int. J. Neuropsychopharmacol..

[118] Xu Y., Zhu N., Xu W., Ye H., Liu K., Wu F., Zhang M., Ding Y., Zhang C., Zhang H. (2018). Inhibition of Phosphodiesterase-4 Reverses Aβ-Induced Memory Impairment by Regulation of HPA Axis Related cAMP Signaling. Front. Aging Neurosci..

[119] Wang H., Zhang F.F., Xu Y., Fu H.R., Wang X.D., Wang L., Chen W., Xu X.Y., Gao Y.F., Zhang J.G. (2020). The Phosphodiesterase-4 Inhibitor Roflumilast, a Potential Treatment for the Comorbidity of Memory Loss and Depression in Alzheimer’s Disease: A Preclinical Study in APP/PS1 Transgenic Mice. Int. J. Neuropsychopharmacol..

[120] Hassan G., Kamar S.A., Rady H.Y., Abdelrahim D.S., Abdel Hay Ibrahim N.H., Lasheen N.N. (2024). A study of roflumilast treatment on functional and structural changes in hippocampus in depressed Adult male Wistar rats. PLoS ONE.

[121] Corpas R., Griñán-Ferré C., Palomera-Ávalos V., Porquet D., García de Frutos P., Franciscato Cozzolino S.M., Rodríguez-Farré E., Pallàs M., Sanfeliu C., Cardoso B.R. (2018). Melatonin induces mechanisms of brain resilience against neurodegeneration. J. Pineal Res..

[122] Aminyavari S., Zahmatkesh M., Khodagholi F., Sanati M. (2019). Anxiolytic impact of Apelin-13 in a rat model of Alzheimer’s disease: Involvement of glucocorticoid receptor and FKBP5. Peptides.

[123] Naik S., Katariya R., Shelke S., Patravale V., Umekar M., Kotagale N., Taksande B. (2023). Nattokinase prevents β-amyloid peptide (Aβ(1-42)) induced neuropsychiatric complications, neuroinflammation and BDNF signalling disruption in mice. Eur. J. Pharmacol..

[124] Amani M., Shokouhi G., Salari A.A. (2019). Minocycline prevents the development of depression-like behavior and hippocampal inflammation in a rat model of Alzheimer’s disease. Psychopharmacology.

[125] Esmaeili M.H., Bahari B., Salari A.A. (2018). ATP-sensitive potassium-channel inhibitor glibenclamide attenuates HPA axis hyperactivity, depression- and anxiety-related symptoms in a rat model of Alzheimer’s disease. Brain Res. Bull..

[126] Zabot G.C., Medeiros E.B., Macarini B.M.N., Peruchi B.B., Keller G.S., Lídio A.V., Boaventura A., de Jesus L.C., de Bem Silveira G., Silveira P.C.L. (2024). The involvement of neuroinflammation in an animal model of dementia and depression. Prog. Neuropsychopharmacol. Biol. Psychiatry.

[127] Yang L., Wu C., Li Y., Dong Y., Wu C.Y., Lee R.H., Brann D.W., Lin H.W., Zhang Q. (2022). Long-term exercise pre-training attenuates Alzheimer’s disease-related pathology in a transgenic rat model of Alzheimer’s disease. Geroscience.

[128] Rosa J.M., Pazini F.L., Camargo A., Wolin I.A.V., Olescowicz G., Eslabão L.B., Romero O.B., Winkelmann-Duarte E.C., AL S.R. (2020). Prophylactic effect of physical exercise on Aβ(1-40)-induced depressive-like behavior and gut dysfunction in mice. Behav. Brain Res..

[129] Bashiri H., Enayati M., Bashiri A., Salari A.A. (2020). Swimming exercise improves cognitive and behavioral disorders in male NMRI mice with sporadic Alzheimer-like disease. Physiol. Behav..

[130] García-Mesa Y., Pareja-Galeano H., Bonet-Costa V., Revilla S., Gómez-Cabrera M.C., Gambini J., Giménez-Llort L., Cristòfol R., Viña J., Sanfeliu C. (2014). Physical exercise neuroprotects ovariectomized 3xTg-AD mice through BDNF mechanisms. Psychoneuroendocrinology.

[131] Ismail Z., Guan D.X., Babulal G.M., Bateman J.R., Cantillon M., Creese B., D’Antonio F., Fischer C.E., Gatchel J.R., Ghahremani M. (2026). Plasma biomarkers in neuropsychiatric syndromes: A narrative review. J. Alzheimers Dis..

[132] Ghahremani M., Leon R., Smith E.E., Ismail Z. (2025). Exploring the association between mild behavioral impairment and plasma p-tau217: Implications for early detection of Alzheimer’s disease. Alzheimers Dement..

[133] Angelopoulou E., Papageorgiou S., Papatriantafyllou J. (2026). Reframing Dementia Prevention Strategies Aligned with the WHO Global Action Plan: A Structured Narrative Review Focusing on Mild Behavioral Impairment. Neurol. Int..

[134] Eratne D., Kang M.J.Y., Lewis C., Dang C., Malpas C., Ooi S., Brodtmann A., Darby D., Zetterberg H., Blennow K. (2024). Plasma neurofilament light outperforms glial fibrillary acidic protein in differentiating behavioural variant frontotemporal dementia from primary psychiatric disorders. J. Neurol. Sci..

[135] Katisko K., Cajanus A., Jääskeläinen O., Kontkanen A., Hartikainen P., Korhonen V.E., Helisalmi S., Haapasalo A., Koivumaa-Honkanen H., Herukka S.K. (2020). Serum neurofilament light chain is a discriminative biomarker between frontotemporal lobar degeneration and primary psychiatric disorders. J. Neurol..

[136] Zhu N., Santos-Santos M., Illán-Gala I., Montal V., Estellés T., Barroeta I., Altuna M., Arranz J., Muñoz L., Belbin O. (2021). Plasma glial fibrillary acidic protein and neurofilament light chain for the diagnostic and prognostic evaluation of frontotemporal dementia. Transl. Neurodegener..

[137] Foiani M.S., Woollacott I.O., Heller C., Bocchetta M., Heslegrave A., Dick K.M., Russell L.L., Marshall C.R., Mead S., Schott J.M. (2018). Plasma tau is increased in frontotemporal dementia. J. Neurol. Neurosurg. Psychiatry.

